# More legislation, more violence? The impact of Dodd-Frank in the DRC

**DOI:** 10.1371/journal.pone.0201783

**Published:** 2018-08-09

**Authors:** Nik Stoop, Marijke Verpoorten, Peter van der Windt

**Affiliations:** 1 Department of Economics, Centre for Institutions and Economic Performance, University of Leuven, Leuven, Belgium; 2 Research Foundation Flanders, Brussels, Belgium; 3 Southern Africa Labor and Development Research Unit, University of Cape Town, Cape Town, South-Africa; 4 Institute of Development Policy, University of Antwerp, Antwerp, Belgium; 5 Division of Social Science, New York University Abu Dhabi, Abu Dhabi, UAE; 6 Development Economics Group, Wageningen University, Wageningen, the Netherlands; Rice University, UNITED STATES

## Abstract

The Dodd Frank Act was passed by the US Congress in July 2010 and included a provision—Section 1502—that aimed to break the link between conflict and minerals in the Eastern Democratic Republic of Congo. To date there is only one rigorous quantitative analysis that investigates the impact of Dodd-Frank on local conflict events. Looking at the short-term impact (2011–2012), it finds that the policy backfired. This study builds on a larger, more representative, dataset of mining sites and extends the time horizon by three years (2013–2015). The results indicate that the policy also backfired in the longer run, especially in areas home to gold mines. For territories with the average number of gold mines, the introduction of Dodd-Frank increased the incidence of battles with 44%; looting with 51% and violence against civilians with 28%, compared to pre-Dodd Frank averages. Delving deeper into the impact of the conflict minerals legislation is important, as President Trump suspended the legislation in February 2017 for a two-year period, ordering his administration to replace it with another policy.

## Introduction

For many countries, natural resources are a curse rather than a blessing (e.g. [1–6]). In the Democratic Republic of Congo (DRC), untapped deposits of raw minerals are estimated to be worth US$24 trillion [7]. The majority of its population, however, is dismal poor, mainly because both war and political mismanagement have ravaged the country. Conflicts in the DRC, the argument often goes, center around the illegal exploitation of minerals, creating competition between rapacious rebel groups and providing them with the means to purchase weapons and attract fighters [8–10]. To end the ongoing violence, it was deemed necessary to end the illegal trade in natural resources. In this spirit, section 1502 of the Dodd-Frank Wall Street Reform and Consumer Protection Act (“Dodd-Frank” from now onwards) was passed in US legislation in July 2010. Section 1502 requires all companies listed on the US stock market to trace the minerals used in their supply chain and to declare whether these minerals are conflict-free or not. It specifically targets resources from the DRC and focuses on four minerals: tin, tantalum, tungsten–often referred to as the 3Ts–and gold.

When President Trump’s draft executive order to temporarily suspend Section 1502 was leaked by the Guardian on February 8, 2017 [11], it caused commotion and triggered various reactions. The Congolese Minister of Mines stated that the suspension of Section 1502 “*in the long run*, *will jeopardize the stability and security of the DRC by encouraging an escalation in the activities of non-state armed groups*” [12]. Human rights activists, who were among the main actors lobbying for the regulation, also deplored the suspension, calling it *“a gift to predatory armed groups seeking to profit from Congo’s minerals as well as a gift to companies wanting to do business with the criminal and the corrupt*” [13]. Congo scholars, on the other hand, said that Trump was *“right on Congo’s minerals*, *but for all the wrong reasons”*, with the wrong reasons including high compliance costs for American companies [14]. The right reasons, instead, relate to the local back-firing of the conflict minerals legislation. Not only did the legislation lead to a de facto ban on artisanal mining that deprived hundreds of thousands of artisanal mining communities from their livelihoods [8,10,15–17], it was argued that the legislation also failed to address the root causes of the violence. In September 2014, in an open letter, a group of 70 academics and experts wrote that the “*conflict minerals campaign fundamentally misunderstands the relationship between minerals and conflict in the Eastern DRC*” [18]. The misunderstanding is twofold. First, although minerals play a role in the continuation of existing conflicts, they are not its root cause. It is estimated that only 8% of all conflicts are over natural resources [8,19]. Other factors include longstanding political and economic grievances and disputes over the control of land and trade routes [8,20]. Second, since armed groups are engaged in a diverse range of income-generating activities, their existence does not depend on access to mineral revenues [20]. For instance, the United Nations ([21]: cited in [10]) report that, after Dodd-Frank, some armed groups looked for alternative sources of income, including the trade in charcoal, cannabis and palm oil.

From a policy perspective, it is important to get a better understanding of the impact of Dodd-Frank. However, to date there is only one rigorous quantitative analysis that investigates the impact of Dodd-Frank on local conflict events. Parker and Vadheim [22]–‘PV’ from now onwards–make use of geo-referenced data on artisanal mining sites and local conflict from 2004 through 2012 to compare the incidence of conflict before and after Dodd-Frank and between those areas in Eastern Congo affected by the ban and those unaffected. They show that the legislation increased looting of civilians and shifted battles between armed groups from 3T mining areas towards unregulated gold mining areas. PV argue that these findings are consistent with the breakdown of a stationary bandit equilibrium. The argument goes as follows: Section 1502 implied a de facto embargo on 3T but not on gold, which is much easier to smuggle compared to the bulkier 3T minerals. As a result, armed groups stationed at artisanal 3T mines found it more profitable to switch their efforts to looting civilians, and to fight rival groups for the ‘right’ to station at gold mines. At the same time, some armed groups that were stationed at gold mines switched to looting civilians in order to avoid battles with competing groups.

This study builds on PV and makes three contributions. The first contribution relates to data. PV make use of a database on the location of 659 artisanal mining sites in Eastern Congo. The data was collected by the International Peace Information Service (IPIS) between 2008 and 2010, and was available on the IPIS website in June 2012. The IPIS dataset has since been significantly updated. New data collections took place in 2013, 2014 and 2015. The updates more than tripled the number of artisanal mining sites in the dataset to 2,282, greatly increasing the geographic coverage and thus the representativeness of the mapping exercise. The additional sites were established before 2004, but had not been visited prior to 2012 for logistical and security reasons [23]. As such, the PV sample may have been biased towards more accessible mining sites, which are more likely to be affected by legislation compared to mines operating ‘under the radar’. The updated IPIS dataset thus calls for a replication of the PV study.

The second contribution relates to scope. PV focus their analysis on two outcomes of interest: looting of civilians and battles between armed actors. We revisit these outcomes, and additionally look at violence against civilians and riots. We explore violence against civilians because we are interested in the local conflict faced by average Congolese citizens. We further investigate if the Dodd-Frank legislation translated into riots, which are defined as potentially violent demonstrations, often involving a spontaneous action by unorganized, unaffiliated members of society. As indicated above, the de facto ban on artisanal 3T mining strongly affected the livelihoods of many individuals. In Congo, up to 16 percent of the country’s population is dependent on artisanal mining [24]. It has been argued that the DRC government used the ‘conflict minerals’ narrative underlying the Dodd-Frank legislation to allow industrial mining companies to relocate artisanal miners and take control over their concessions [16]. For instance, shortly after the introduction of Dodd-Frank, on September 11, 2010, president Kabila announced a governmental ban on artisanal mining [25,26]. The ban lasted until March 10, 2011 and covered the Kivu provinces and Maniema. Officially, the objective of the ban was to address the issue of ‘conflict minerals’. Some scholars argue, however, that the government really wanted to address “*the fact that artisanal miners are working anywhere*, *including in industrial concessions; the fact that they have no official permits*, *are not organized and not officially registered; and the fact that minerals are being exported without an official export license*” ([16], p.326). These actions further affected the livelihoods of artisanal mining communities and added to the already palpable tension between mining communities and companies. This tension, built up since the introduction of the 2002 Congolese Mining Code and the related promotion of industrial mining, often erupts in riots and violent confrontations (e.g. [27,28]).

The third contribution relates to the time horizon. PV’s data stretched only two years into the de facto ban. This study adds three years, exploring the 2004 to 2015 period. Exploring the longer-term impacts of Dodd-Frank is important, as one could argue that the short-term negative effects of the policy may be offset by longer term gains. For instance, while the policy could indeed cause a disruption and greater violence in the short run, in the longer run, the purchase of weapons may be compromised if revenues of armed groups decline because of the de facto ban, potentially leading to a decrease in violent conflict [22]. In addition, over time, US companies could restructure their global supply chain, organizing so-called closed pipelines, or a black market in ‘conflict minerals’ could develop; both of which could nullify the de facto ban, and its associated (un)intended consequences. If the negative effects of the policy persist over a five-year period, however, any long-term gains would have to be very large to offset the costs. Moreover, our exploration of the longer-run effects is a timely contribution. In Trump’s leaked draft order, the Secretary of State and Secretary of the Treasury were asked to propose an alternative plan for addressing human rights violations and the funding of armed groups in the DRC.

## Context: Minerals, conflict and Dodd-Frank

In this section, we briefly introduce the nexus between minerals and conflict in Eastern Congo, and provide background information on the Dodd-Frank legislation that was expected to sever this relationship.

### Minerals and conflict in Eastern Congo

Eastern Congo was the scene of the first and second Congo wars (1996–1997 and 1998–2003). The First Congo war succeeded in overthrowing the dictatorship of Mobutu Sésé Seko. The initial objective of the second war was to overthrow President Laurent Désiré Kabila, but the various African countries and rebel groups involved also developed their own objectives, which included the appropriation of mineral wealth. President Laurent Désiré Kabila was assassinated in 2001 but was succeeded by his son Joseph Kabila. Both wars are described and discussed at length by, amongst others, Autessere [29], Reyntjens [30] and Stearns [31].

Despite the formal end of the Second Congo War, a national unity government in 2003, and general elections in July 2006, violence continued. In 2010, the year in which the Dodd-Frank legislation passed, over a dozen armed groups were active in Eastern Congo, and approximately 1.7 million people remained displaced [32]. Several factors explain the continued violence. For instance, due to the infusion of arms and rebels, many dormant local conflicts turned violent. These local conflicts were not addressed in the peace accords that instead focused on national and international issues [8]. Furthermore, Kabila junior’s rule proved inapt to turn the war logic around and transit to a peace economy. In the words of Stearns and Vogel “*the government and its foreign partners have been unable to create a virtuous cycle of economic development in the rural Kivus that could entice local leaders to invest in stability rather than conflict*” ([33], p.8). The availability of easy access to resources is further seen as a major contributor to the continuation of the violence, as documented among others by the reports of the UN Group of Experts on the DRC [34].

The ‘conflict minerals’ of Eastern Congo comprise the so-called 3Ts (tin, tungsten, tantalum) and gold. In roughly half of these minerals’ artisanal mining sites, armed actors are present on a permanent or regular basis [23]. These armed actors, including both Congolese and foreign rebel groups as well as the Congolese army (FARDC), mostly profit from artisanal mining through illegal taxation, but are also known to engage in mineral trade, to monopolize the sale of certain commodities (e.g. beer, cigarettes or palm oil), force artisanal miners to work for them, or resort to looting and pillaging [10,20,23,35].

### Dodd-Frank and the DRC mining ban

In an attempt to break the cycle of violence, several advocacy groups, most prominently the Enough Project and Global Witness, lobbied for policies that would cut the revenue stream from minerals to armed groups. The lobbying efforts culminated in Dodd-Frank’s Section 1502, which—in the words of one of its promotors—was aimed at *“reducing the size of the black market and intended to reduce the funding of violence while making progress on governance*, *peace*, *and security issues more possible”* ([36], p.184). In particular, Section 1502 required all companies listed on the US stock market to determine the exact origin of minerals sourced from conflict areas and to reveal their supply chains to the US Securities and Exchange Commission. While the legislation did not include any legal penalties for non-compliance, it entailed the risk of brand damage following public naming and shaming [15].

Instead of running this risk and going through the uncertain, costly and complex process of documenting the entire supply chain, many companies simply decided to no longer purchase minerals from the conflict zones mapped by the US State Department. After all, the DRC only supplies a tiny fraction of the world supply of gold, tungsten and tin; and while an important supplier of coltan (at an estimated 15% of the total world production) [37,38], there remained sufficient outside options. To everyone’s surprise, this de facto ban was pre-empted by a governmental ban imposed by President Kabila on all artisanal mining activities in three provinces in Eastern Congo–Maniema, North Kivu, and South Kivu. The ‘Kabila embargo’ was in force from 9 September 2010 till 10 March 2011. Before the end of this de jure embargo, however, it became clear that it would be succeeded by a de facto embargo, when two global coalitions of major electronic companies announced that they stopped buying minerals from smelters who couldn’t prove that they did not source minerals that fund conflict in the DRC [10,15,39]. The two coalitions are the Electronic Industry Citizenship Coalition (EICC–which includes a.o. Apple, HP, Dell and Microsoft), and the Global e-Sustainable Initiative (GeSI–which includes a.o. Motorola and Nokia). Because of this decision, the Malaysia Smelting Corporation (MSC), which previously purchased up to 80% of Eastern Congolese tin, stopped sourcing minerals from the DRC [10,15].

Not surprisingly, official export data reveal a large drop in exports of tin, coltan (tantalum), and wolframite (tungsten) during 2010–12, suggesting that the de facto ban indeed negatively impacted 3T mining activities [22]. Official data may however not be reliable, as the ban may have induced smuggling. While smuggling is highly likely in the case of easy-to-conceal gold, it is much less straightforward in the case of bulky 3T; the more so because 3Ts come with waste rock that has to be removed in smelting facilities. Furthermore, the effective slowdown of 3T mining is confirmed by satellite images that show a slowing of deforestation around 3T mining sites, but not around gold sites [22]. It should be noted that although mining activities slacked because of the embargo, mineral trade did not stop entirely. First, as was the case before the introduction of Dodd-Frank, gold was smuggled across the DRC’s eastern borders [24,40,41]. Second, Chinese buyers, who were not affected by Dodd-Frank, continued to export 3T minerals from the DRC, be it at a very large discount ([40], p.105).

There is a general consensus that the slowdown of mining activities had an immediate negative effect on living conditions of artisanal miners and the interlinked local economy [8,10,15–17]. These effects are also recognized by the proponents of Section 1502, but they are portrayed as a necessary evil for a greater good, namely the reduction of the black market in minerals, and its assumed stabilizing effect [36]. Advocacy groups have further claimed that the Dodd-Frank legislation was successful, based on the argument that a reduction in revenues from 3T mining leads to a reduction in the financing and strength of armed groups [42]. Many scholars, on the other hand, have argued that Dodd-Frank has done little to improve the security situation in Eastern Congo, and that armed groups have looked for alternative sources of income, including the trade in charcoal, cannabis and palm oil [10]. Moreover, PV demonstrate that “*the [Dodd-Frank] legislation increased the probability of civilian looting by at least 143% and that it increased the probability of battles in territories endowed with unregulated gold*” ([22], p.13). These outcomes can be considered as so-called crime displacement effects, a phenomenon that has been widely documented in the crime and conflict literature (e.g. [43–45]).

In addition, scholars have argued that increased insecurity has come from unemployed miners, who turned to criminal activities in order to provide for a living. Geenen, for instance, mentions an *“increased incidence of thefts*, *robberies*, *armed attacks and murders during the ban*, *because of a generalized deterioration of the economy and rising levels of unemployment”* ([16], p.327). The assumed mechanism here is akin to the opportunity cost mechanism, inspired by Becker’s cost-benefit approach to the analysis of crime [46], and widely referred to in the literature on civil war [47–51]. Simply put: the negative shock to the mining sector decreased the miners’ opportunity cost, drawing labor into criminal and rebel activities.

### Long-term impact of Dodd-Frank?

To date, quantitative evidence related to the impact of Dodd-Frank is largely based on assessments of the short-term impact of the legislation; up to two years after the ban in the case of PV. Defenders of Dodd-Frank might claim that its ultimate aim *“to reduce the funding of violence while making progress on governance*, *peace*, *and security issues more possible”* ([36], p. 184) could yet have been achieved in the longer run. The argument for such a long-term effect could go as follows: The crime displacement effect cannot entirely substitute for the foregone revenue stream from 3T mining. Consequently, over time, armed groups will face problems financing labor and capital, and will lose weapon- and man-power. This gives room for the state to move in the power vacuum, and establish law and order, which in its turn allows the local economy to recover from the initially negative shock, bending the opportunity cost effect again in favor of more peaceful activities.

While possible in theory, this scenario is unlikely in the current economic and political climate in DRC. First, the ‘conflict minerals’ legislation has not addressed the root causes of local conflict. While minerals provide fuel for the conflict, its root causes include disputes over land and political power, and these remain poorly understood and unaddressed in the various peace accords [29]. Second, while it is right to assume that the Congolese state may move in the power vacuum, it is wrong to assume that the kind of law and order it establishes will be to the benefit of local communities. Geenen discusses for instance how the ban merely shifted the illicit control of mining sites from rebels or disloyal army units towards politico-military authorities more loyal to Kabila, and how it allowed industrial concessions (unaffected by the ban) to expel artisanal miners from their concessions [16]. The ban thus shifted power from local elites to national elites in Kinshasa, and from small-scale actors to industrial companies, and their promotors in Kinshasa.

As long as these fundamental causes, i.e. unresolved local grievances and a predatory state, are not addressed in a more systemic approach, any narrowly defined ‘conflict minerals’ legislation may come short of reaching its ultimate objective of restoring social and economic order. This point of view is in line with the conclusions drawn in the literature on economic sanctions. First, in general, sanctions have a poor track record in terms of their impact on a country's economy [52–54]. Second, there are very few cases where sanctions have had success, and when they had, it was in combination with other factors [55–58]. That sanctions are still a popular policy tool given this poor track record, is to a large extent because of their symbolic value, which–at times–is principally used to appease domestic constituencies, or to make a moral and historical statement [55,58–60].

In the remainder of this study, we explore the impact of Dodd-Frank on local level conflict in Eastern Congo.

## Data

**Fig 1:**
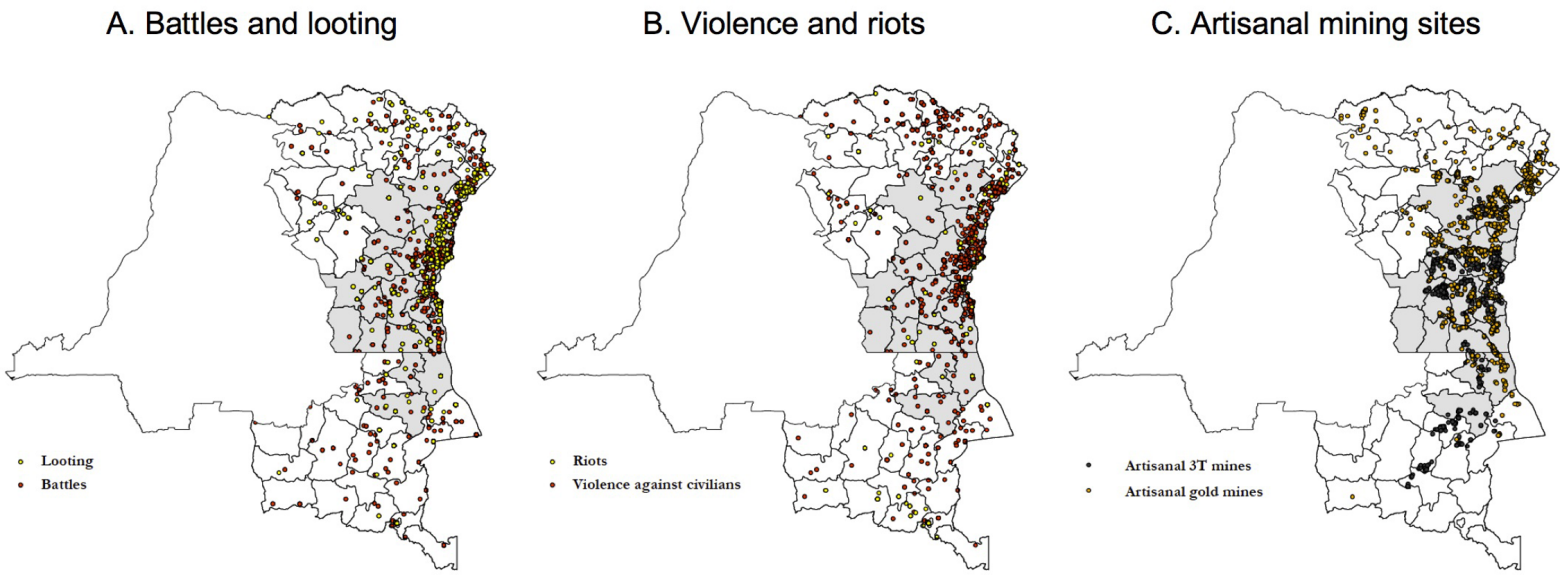
Maps. Panel (a) shows the location of battles and looting events that occurred between 2004 and 2015. Panel (b) shows the location of violence against civilians and riots. Panel (c) shows the location of artisanal 3T and gold mines. Shaded territories constitute the Dodd-Frank treatment area.

Our analysis focuses on Eastern Congo, which is subdivided in 70 administrative territories (see **Fig 1**). To identify the impact of Section 1502 of the Dodd-Frank act–also indicated as ‘DF’ from now onwards–we rely on geo-referenced information of conflict events and mining sites for the period 2004–2015. We further use data on mineral prices and rainfall. This section describes the data in detail. Summary statistics are available in **Table 1**.

**Table 1:** Summary statistics.

Variable	Mean	SD	Min	Max	Description
*Panel A*: *Time varying variables*:					
Dodd-Frank indicator ^a^	0.18	0.38	0	1	= 1 from July 2010 onwards for territories that are located within the Dodd-Frank treatment area.
Looting indicator ^b^	0.04	0.19	0	1	= 1 if there was at least one conflict event described with the words: loot, pillage, plunder, rob, steal, ransack or seize. Indicates the action of an armed group against civilians.
Battles indicator ^b^	0.11	0.31	0	1	= 1 if there was at least one battle event. Battles are violent interactions between two organized armed groups at a particular time and location.
Violence against civilians indicator ^b^	0.11	0.32	0	1	= 1 if there was at least one event of violence against civilians; occurs when an armed group attacks civilians.
Riots indicator ^b^	0.04	0.19	0	1	= 1 if there was at least one riot. Riots are potentially violent public demonstrations by groups.
Gold price ^c^	2.48	1.00	0.93	4.28	International gold price, normalized at 1 based on the January 2004 price.
Tin price ^c^	1.90	0.72	0.71	3.72	International tin price, normalized at 1 based on the January 2004 price.
Tungsten price ^c^	4.20	1.18	1.00	6.35	International tungsten price, normalized at 1 based on the January 2004 price.
Tantalum price ^c^	1.95	0.96	0.89	3.46	International tantalum price, normalized at 1 based on the January 2004 price.
Rainfall anomaly ^d^	-0.01	0.87	-4.25	4.10	Monthly deviation from the 1997–2015 monthly mean, divided by the 1997–2015 SD.
Adjacent conflict indicator ^b^	0.35	0.48	0	1	= 1 if there was any conflict event (looting, battles, violence against civilians, riots) in an adjacent territory.
*Panel B*: *Time invariant variables*:					
Gold mines (PV) ^e^	5.79	11.03	0	69	# gold mines in PV set-up
Tin mines (PV) ^e^	2.91	6.37	0	33	# tin mines in PV set-up
Tantalum mines (PV) ^e^	0.31	1.21	0	9	# tantalum mines in PV set-up
Tungsten mines (PV) ^e^	0.11	0.62	0	5	# tungsten mines in PV set-up
Gold mines (full) ^e^	22.93	42.52	0	193	# gold mines in full sample of mines
Tin mines (full) ^e^	7.43	15.97	0	72	# tin mines in full sample of mines
Tantalum mines (full) ^e^	1.80	5.71	0	39	# tantalum mines in full sample of mines
Tungsten mines (full) ^e^	0.44	1.31	0	7	# tungsten mines in full sample of mines
Dry month indicator ^d^	0.25	0.43	0	1	= 1 for the three driest months in each territory, based on 1997–2015 precipitation averages.
Wet month indicator ^d^	0.25	0.43	0	1	= 1 for the three wettest months in each territory, based on 1997–2015 precipitation averages.

**Notes:** *N* = 10,080 –with 144 monthly observations (Jan. 2004 –Dec. 2015) for 70 territories.

^a^ The 27 treatment territories are shaded in **Fig 1**.

^b^ Data on conflict variables comes from ACLED, the Armed Conflict Location and Event Data project. Battles, violence against civilians and riots are coded by ACLED. We follow PV in coding looting events as indicated in Table 1.

^c^ Data on mineral prices was obtained from metalprices.com.

^d^ Climatic information comes from CRU, the Climatic Research Unit of the University of East-Anglia.

^e^ Data on mining sites comes from IPIS, the International Peace Information Service. In Eastern Congo, tin is derived from the ore cassiterite; tantalum from coltan and tantalite; and tungsten from wolframite.

### Dodd-Frank treatment

We create an indicator variable that assigns ‘DF treatment’ over time and across territories. We follow PV in defining the treatment area based on the intersection of territories that were impacted by the DRC governmental ban on artisanal mining (i.e. all 22 territories of North-Kivu, South-Kivu and Maniema) and the territories on the US State Department’s Section 1502 map of conflict mining zones (which additionally includes three territories in Katanga and two territories in Orientale). These 27 treatment territories are shaded in **Fig 1**. Following PV, the treatment starts in July 2010, when DF was passed in US legislation. The remaining territories plus the territory-month observations in treatment territories before July 2010 are assigned to the control.

### Local conflict outcomes

To measure local conflict, we build on the Armed Conflict Location and Event Data Project (ACLED). ACLED provides information on the date and location of conflict events in 60 developing countries in Africa and Asia. It mainly relies on reports from local and regional news sources, as well as humanitarian agencies [61], and has been widely used in recent academic research [22,62–64].

For Eastern DRC, ACLED contains information on the date and location of 6,542 conflict events that occurred between 2004 and 2015. The database allows us to separate out the type of conflict. We focus on four different types, of which three (battles, violence against civilians and riots) are directly coded by ACLED. First, battles are defined by ACLED as *“a violent interaction between two politically organized armed groups at a particular time and location”* [65], where armed groups include both rebels and the Congolese state army (FARDC). The database contains 2,748 battle events. Second, violence against civilians occurs when armed groups attack civilians. The database contains 2,487 such events. Third, riots are coded as potentially violent public demonstrations by groups. We have information on 518 riot events. Finally, we follow PV and use the description of the conflict events to construct a variable that indicates events of looting. We consider an event as looting if an armed group’s actions against civilians are described by the words ‘loot’, ‘pillage’, ‘plunder’, ‘rob’, ‘steal’, ‘ransack’, ‘sack’, or ‘seize’. Examples of looting include: “Rebels loot 10 houses in Kiwanja and 3 others in Rubare and Kako”; “FDLR rebels established a base for looting gold and cassiterite from the mines at Kasiyiro”; and “Soldiers erected illegal barriers at Mangi and Panga mining sites in Banalia area since the beginning of June, extorting and seizing goods from mine workers and merchants”. In total, we have information on 718 looting events.

Panels (a) and (b) of **Fig 1**indicate the location of these conflict events in Eastern Congo. The large majority of territories have witnessed them at least once over the period of study: looting (70%), battles (90%), violence against civilians (97%) and riots (79%); while for some territories, they occurred during a large number of months (the maxima are 32 for looting, 87 for battles, 79 for violence against civilians and 44 for riots). In **Table 1**, we report the territory average monthly incidence of looting (4%), battles (11%), violence against civilians (11%) and riots (4%).

### Mining sites

Information on mining sites comes from the International Peace Information Service (IPIS). PV make use of a database on the location of 659 artisanal mining sites, which was collected between 2008 and 2010, and was available on the IPIS website in June 2012. The IPIS dataset has since been significantly updated. New data collections took place in 2013, 2014 and 2015 in which IPIS partnered up with the Congolese Ministry of Mines, other Congolese mining services and representatives from local civil society organizations. The update more than tripled the number of artisanal mining sites compared to PV, to 2,282. Importantly, the increase in mining sites reflects the expanded geographic coverage of the mapping exercise rather than an increase in mining activities [23]. Available information indicates that all mines in the full database were established before 2004 and existed throughout the entire period of study, but many were not visited before for logistical and security reasons (for further discussion see S1 Appendix). The data and collection process of the different rounds are described in detail in various IPIS reports [23,66,67].

**Fig A** in S1 Appendix illustrates the location of the artisanal mining sites in the PV set-up and the updated database. **Table 1**shows summary statistics for both samples of mining sites. In the full sample, 71% of territories contain artisanal mining sites; 39% have 3T mines and 61% have gold mines. The number of 3T mines in a territory varies between 0 and 78, while the number of gold mines varies between 0 and 193.

### Mineral prices

**Fig 2:**
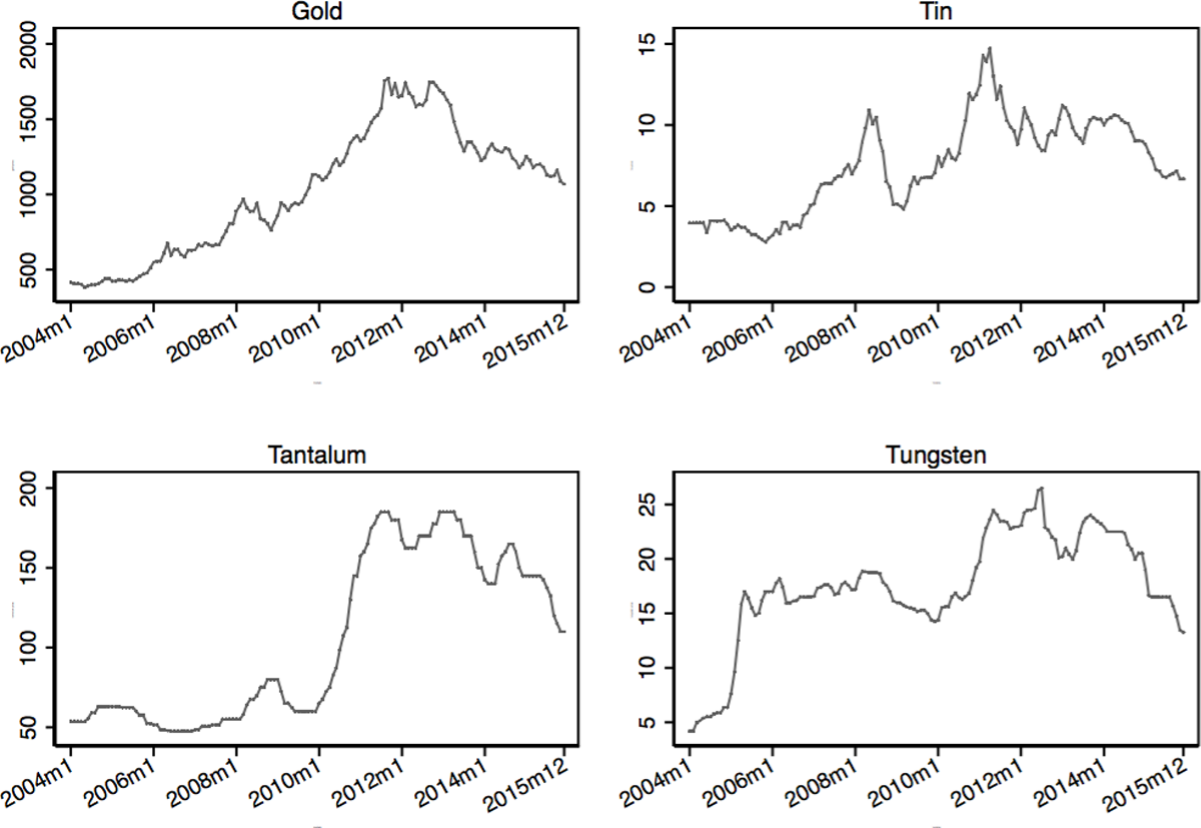
World prices of gold and 3T minerals. Notes: These graphs show the monthly averages of world prices for gold, tin, tantalum and tungsten for the period 2004–2015 in 2015 US dollars. Gold prices are reported in U.S. dollars per troy ounce. 3T prices are reported in U.S. dollars per pound. The data was obtained from metalprices.com.

We obtain time-series data on international mineral prices from metalprices.com. **Fig 2**shows the monthly average prices for gold and the 3T minerals over the period 2004–2015. Gold prices are reported in dollars per troy ounce; 3T prices are reported in dollars per pound. In the empirical analysis we normalize mineral prices to 1 based on the January 2004 price. Summary statistics are presented in **Table 1**. Over the period of our study, world mineral prices of gold and 3T minerals on average tripled. For instance, in constant 2015 US dollars, a troy ounce of gold was on average valued at 326$ in 2004, while it was worth 1,160$ in 2015. This boom in commodity prices is generally explained by an increasing demand in emerging economies, particularly China [68,69]. The increase in world mineral prices of gold and 3T averages about two standard deviations over the period of our study. We do not have detailed information on local mineral prices. Fieldwork by Geenen [35], however, indicates that local mineral traders in Eastern Congo closely monitor world mineral prices and use them to set local prices (for further discussion see S2 Appendix).

### Rainfall

We follow a number of recent papers [22,63,70] and control for rainfall as a proxy for exogenous shocks to agricultural income. On the one hand, an increase in agricultural income may raise the opportunity cost to join armed groups. On the other hand, it may increase armed groups' incentives to loot farmer communities. Heavy rainfall could also hinder mining activities and the movement of armed groups.

We use monthly rainfall data from the Climatic Research Unit of the University of East Anglia. The data is available at a spatial resolution of approximately 55 x 55 kilometers. We calculated average monthly rainfall values for each territory. First, we follow Maystadt et al. [63] in calculating rainfall anomalies; these measure deviations from normal rainfall conditions for each territory-month observation. Specifically, the anomalies measure the monthly deviation from the long-term monthly mean, divided by the monthly long-term standard deviation. Second, to control for the possibility that seasonal patterns may matter, we follow the example of PV in constructing variables to indicate wet and dry seasons. Based on the long-run monthly rainfall averages, we create two dummy variables that indicate the three driest and the three wettest months for each territory. **Table 1**shows summary statistics.

## Empirical analysis

Our empirical strategy follows PV. To identify the impact of Section 1502 of DF on local conflict events, we compare the incidence of conflict over time (before and after DF) and across territories (those affected by DF and those unaffected). The DF treatment starts in July 2010. The 27 treatment territories are shaded in **Fig 1**. The identifying assumption is the parallel trends assumption: in the absence of treatment, violence in the treated territories would have followed a trend that is parallel to the trend of violence in the non-treated territories. Before moving to the econometric analysis, we present graphical evidence on the evolution of conflict events through time and across treated and non-treated territories.

**Fig 3:**
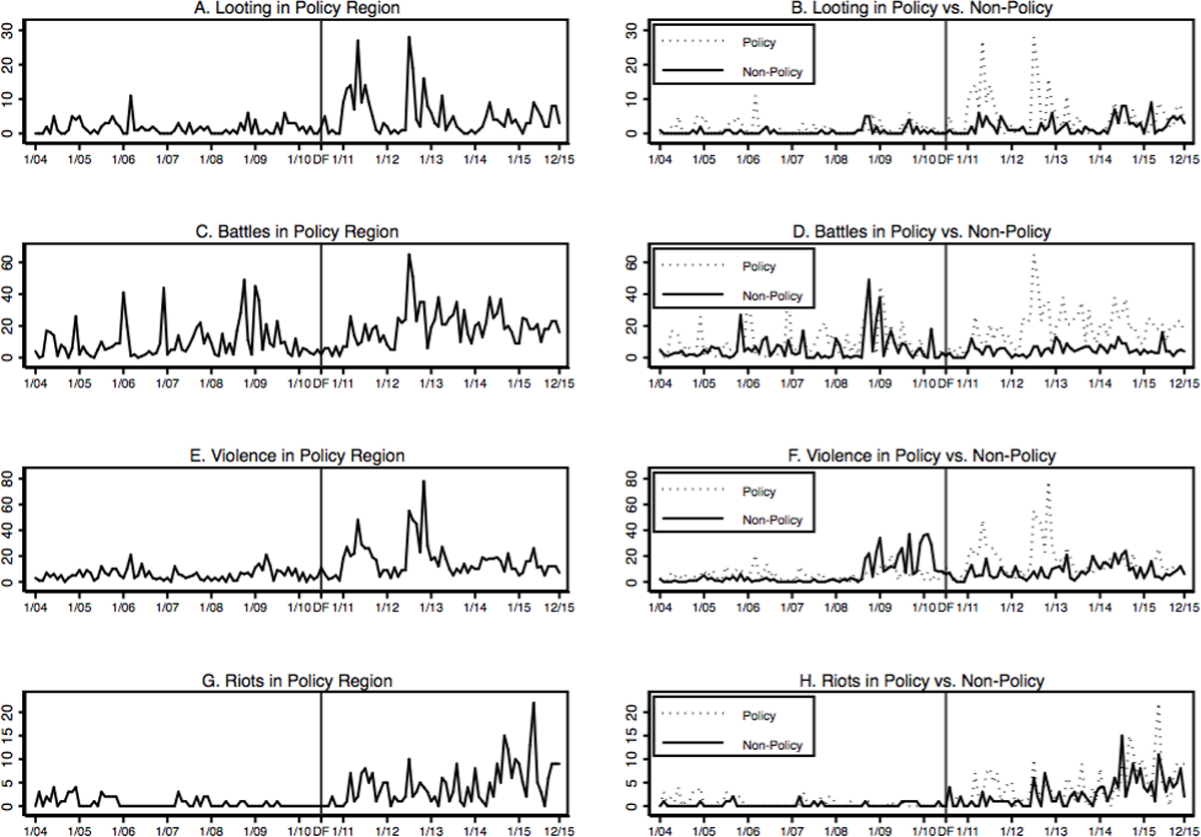
Monthly conflict events 2004–15. Notes: These graphs show the average monthly number of looting, battles, violence and riots events for the period 2004–15. The treatment area and conflict measures are described in the section ‘Data’. The vertical line indicates the start of the Dodd-Frank treatment.

**Fig 3**shows the average monthly number of looting, battles, violence against civilians and riots events for the period 2004–15. The graphs in the first column display the evolution of conflict events in treated territories. After the introduction of DF, all types of conflict events increased compared to the period before; most clearly so for looting, violence against civilians and riots (Panels A, E and G). The graphs do not show evidence of an upward trend in conflict events in the period just preceding the introduction of DF. The second column of **Fig 3**compares the evolution of conflict events across treated and non-treated territories. While there is no one-on-one relationship, overall, we find similar pre-Dodd-Frank trends in looting, battles, violence against civilians and riots. After the introduction of Dodd-Frank, however, we see that the number of conflict events increased in treated territories compared to non-treated ones. This is especially clear for battles (Panel D), and in the period 2010–12 also for looting and violence against civilians (Panels B and F). We now move to an econometric analysis in order to control for potentially confounding covariates.

### Econometric model

The econometric specification is given by:

$${\text{conflict}}_{it}=\delta_{i}+\mu_{t}+\beta_{1}{\text{DF}}_{it}+\beta_{2}\left({\text{DF}}_{it}\times 3T_{i}\right)+\beta_{3}\left({\text{DF}}_{it}\times {\text{gold}}_{i}\right)+\overset{4}{\underset{m=1}{\sum }}\lambda_{m}\left({\text{mine}}_{im}\times {\text{price}}_{tm}\right)+\overset{3}{\underset{k=1}{\sum }}\gamma_{k}{\text{season}}_{i}+\overset{2}{\underset{x=0}{\sum }}\eta_{x}{\text{rain}}_{i,t-x}+\overset{3}{\underset{x=1}{\sum }}\alpha_{x}{\text{conflict}}_{i,t-x}+\overset{1}{\underset{x=0}{\sum }}\emptyset_{x}\text{adj}.{\text{conflict}}_{i,t-x}+\varepsilon_{it}$$ (1),

where i denotes the territory, t denotes the month, m denotes the four minerals (gold, tin, tantalum, tungsten), and k denotes the season (dry, wet or neither). The dependent variable, conflict_*it*_, denotes the monthly incidence of looting, battles, violence or riots in territory i. The territory fixed effects, $\delta_{i}$, control for time-invariant territory-specific determinants of conflict events such as ethnic composition and geography. The month fixed effects, *μ*_*t*_, control for Eastern Congo-wide factors that may cause changes in conflict, such as elections. The coefficients of interest are: *β*_*1*_, *β*_*2*_ and *β*_*3*_. They measure how conflict is affected by the DF-induced de facto ban, and how this impact varies with the number of 3T and gold mines in a territory. We control for monthly changes in the international price of gold and 3T minerals in territories where those minerals are mined (mine_*im*_*×*price_*tm*_); territory-specific seasonal patterns (season_*i*_); and contemporaneous and lagged rainfall anomalies (rain_*i*,*t-x*_).

Conflict may persist through time and across space. We therefore also include lags for the incidence of conflict events in previous months (conflict_*i*,*t-x*_) and in adjacent territories (adj.conflict_*i*,*t-x*_). These dynamic and spatial lags capture the incidence of all types of conflict events, thus taking into account that past battles may affect e.g. future violence against civilians and looting, or the other way around. We follow PV and include three-month lags for within-territory conflict (*x* ∈{0,3}), as well as contemporaneous conflict in adjacent territories and a one-month lag (*x* ∈{0,1}). The results are, however, robust to adding up to twelve-month dynamic and spatial lags (see the section ‘Robustness’). We realize that the coefficients on these lags may be estimated with bias (e.g. [71,72]); we thus only introduce them to check the robustness of our beta coefficients (for further discussion see S3 Appendix). Finally, we apply the methodology developed by Conley [73], and implemented by Hsiang et al. [74], to correct standard errors for both spatial correlation and location-specific serial correlation. Following the example of Berman et al. [62], we present all specifications with a correction that allows for spatial correlation within a radius of 500 km and a practically infinite horizon for serial correlation (100,000 months). The results are robust to alternative specifications for the standard errors (see the section ‘Robustness’).

We estimate Eq (1) using a Linear Probability Model. Our empirical strategy closely follows PV, but differs in three ways. First, we centered the variables indicating the number of 3T and gold mines in a territory, which means that coefficients related to the DF indicator should be interpreted as changes for a treated territory with the average number of 3T and gold mines, rather than for a treated territory without any 3T or gold mines. We do so, because only three out of 27 treated territories have no 3T or gold mines at all. Second, in our main results, we do not include territory specific linear time trends because we believe it is unlikely that conflict events follow a linear pattern, and because adding 70 covariates may create unnecessary noise. We do include them in a robustness check (see the section ‘Robustness’). Third, we present all results with Conley [73] standard errors that have been corrected for spatial and serial correlation.

### Main results

**Fig 4:**
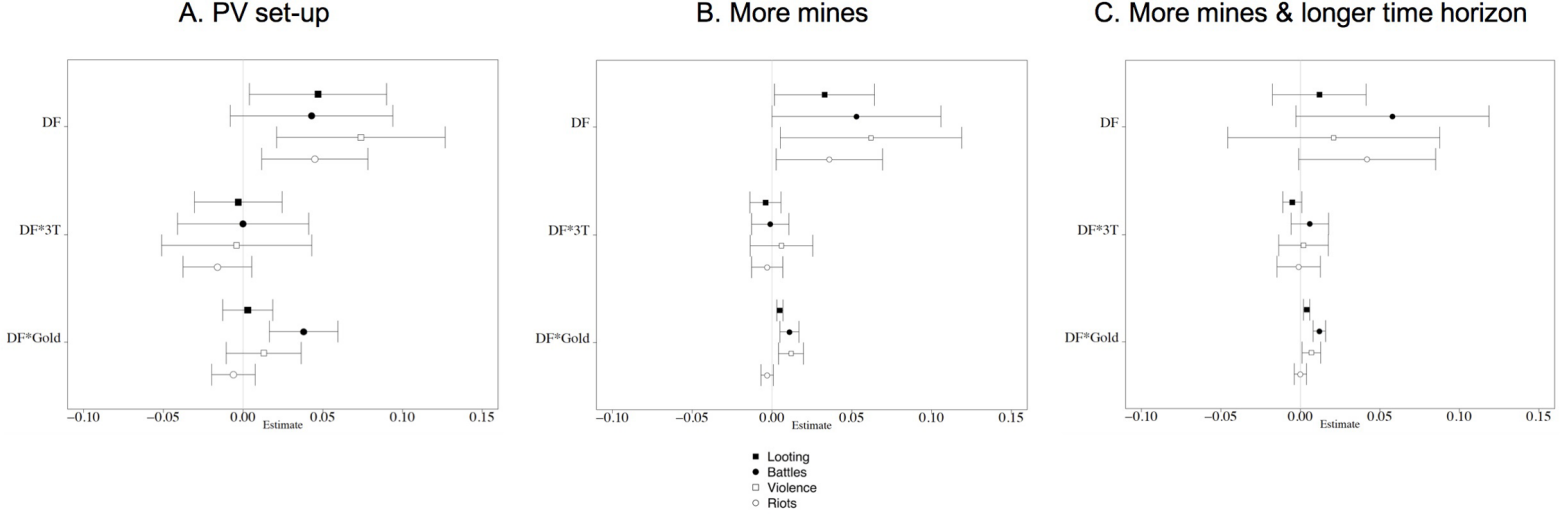
Results. **Notes:** Bars are 95% confidence intervals, based on Conley [73] standard errors allowing for spatial correlation within a 500 km radius and for infinite serial correlation. Panel (a) uses the PV set-up (small sample of mines, short time horizon 2004–2012). Panel (b) uses the full sample of mines. Panel (c) uses the full sample of mines and increases the time horizon to cover the period 2004–2015.

We estimate different specifications of Eq (1), where we start by setting *λ*, *γ*, *η*, α, and ∅ to zero, and then gradually allow these coefficients to take non-zero values. **Fig 4**presents the results related to our variables of interest from the most inclusive specifications. Each dot is a point estimate and bars present 95% confidence intervals. “DF”, “DF*3T” and “DF*Gold” are the estimates of equation’s *β*_*1*_, *β*_*2*_ and *β*_*3*_, respectively. The full set of results, which are robust across model specifications, can be consulted in tabular form in S4 Appendix.

In panel (a) of **Fig 4**, we use the PV set-up (small sample of mines, period 2004–2012). We find that the DF legislation increased looting, battles, violence against civilians and riots in DF-treated territories. Before the introduction of DF, the average monthly probability of conflict in these territories equaled 0.037 for looting, 0.127 for battles, 0.119 for violence against civilians and 0.018 for riots. The coefficient estimates of 0.047, 0.043, 0.074 and 0.045 indicate that the probability of these conflict events increased with 127%, 34%, 62% and 250%. These increases are statistically significant at the 1% significance level for violence against civilians and riots, at the 5% level for looting, and at the 10% level for battles. The DF legislation especially increased the incidence of battles in gold mining areas. The coefficient of 0.038 implies that the monthly probability of battles increased with 3.8 percentage points with every additional ten gold mining sites in a territory. Using the small sample of mines, the average DF territory has 10.4 gold mining sites. Hence, for territories with the average number of gold mines, this result implies an increase of 4 percentage points or 31% compared to the average monthly probability of battles before the introduction of DF.

**Fig 4**‘s panel (b) replicates panel (a) but makes use of the full IPIS database. Again, we find that the DF legislation increased all conflict events in the average DF-treated territory. The coefficient estimates of 0.033, 0.053, 0.062 and 0.036 indicate significant increases in the monthly probability of looting (89%), battles (42%), violence against civilians (52%), and riots (200%) compared to the pre-DF averages. Moreover, the monthly probability of looting, battles and violence against civilians significantly increased with the number of gold mining sites in a territory: for every additional ten gold mining sites, we find an increase of 0.5, 1.1 and 1.2 percentage points respectively. Using the full sample of mines, the average DF territory has 46.8 gold mining sites. Compared to pre-DF averages, and for territories with the average number of gold mines, these results imply an increase of 71% for looting; 41% for battles; and 47% for violence against civilians.

Finally, panel (c) of **Fig 4**presents results where we use the full sample of mines and increase the time horizon after the introduction of DF from two to five years, covering the period 2004–2015. Using this set-up, we find that the DF legislation increased battles and riots in the average DF-treated territory (although these results are only significant at the 10%-level). The coefficient estimates of 0.058, and 0.042 indicate that the monthly probability of battles and riots increased with 46% and 233% compared to the pre-DF averages. We still find that the monthly probability of looting, battles and violence against civilians significantly increased with the number of gold mining sites in a territory. For every additional ten gold mining sites, we find an increase of 0.4, 1.2 and 0.7 percentage points respectively. The longer-run impact of Dodd-Frank is thus concentrated in gold mining areas. Looting, battles and violence against civilians did not significantly increase in territories without gold mines. However, for the average Dodd-Frank territory–with 46.8 gold mines–the results imply an increase of 51% for looting; 44% for battles; and 28% for violence against civilians, compared to pre-DF averages. These increases are even more substantial for DF-territories with the highest concentration of gold mines. The top quarter of territories with most gold mines have between 68 and 193 gold mines, averaging at 128. In these territories, the introduction of Dodd-Frank on average implied an increase of 138% in looting, 135% in battles and 70% in violence against civilians. Finally, the results in panel (c) also indicate that the monthly probability of looting significantly decreased with the number of 3T mines in a territory. For every additional ten 3T mining sites, we find a decrease of 0.5 percentage points (this result is however only significant at the 10%-level, and only in the most inclusive specification–see Table D in S4 Appendix).

### Robustness

Before we move to the conclusion we show that the main results are robust to a series of checks.

A first set of robustness checks deals with spatial correlation. First, we test the robustness of the results to using alternative spatial and temporal specifications when correcting the standard errors (see **Table E** in S5 Appendix). For each estimated coefficient, we present five sets of standard errors: 1) Conley standard errors allowing for spatial correlation within a 1,000 km radius and 100,000 months of serial correlation; 2) Conley standard errors allowing for spatial correlation within a 100 km radius and 100,000 months of serial correlation; 3) Conley standard errors allowing for spatial correlation within a 100 km radius and 5 years of serial correlation; 4) Conley standard errors allowing for spatial correlation within a 100 km radius and 1 year of serial correlation; 5) clustering the standard errors at the level of the territory. The results are highly robust to these alternative specifications. Second, when presenting the main results, we follow PV and include up to three-month lags for within-territory conflict, as well as contemporaneous conflict in adjacent territories and a one-month lag. In **Table F** in S5 Appendix, we control for up to twelve-month dynamic and spatial conflict lags; the results are robust to including these additional lags.

A second set of robustness checks uses alternative conflict measures. First, to explore both the intensive and extensive margin of local violence, we also look at conflict intensity instead of conflict occurrence alone, replacing the monthly indicators for looting, battles, violence against civilians and riot events with variables that indicate the number of conflict events that occurred in a specific territory and month. Second, we combine the four monthly conflict event indicators into one variable that equals one when any of the looting, battles, violence against civilians or riot events occurred. The results are presented in **Table G** and **Table H** in S5 Appendix and follow a similar pattern as the main results presented above. Finally, we note that it is possible that conflict events had more news value after the introduction of Dodd-Frank and were thus more likely to be reported about. While we cannot exclude this possibility, we have no indication for such a change in reporting. Furthermore, even if such a reporting bias exists, it is unlikely that it would entirely drive our results as the bias would have to exist only for conflict events that occurred in the 27 treated territories, and not for conflict events that occurred in the control group.

In a third set of robustness checks we employ an alternative definition of our mining variables. Rather than looking at the impact of DF by the number of 3T and gold mines in a territory, we create a variable that indicates the share of all 3T and gold mines that is located within a specific territory. This variable gives an indication of the relative importance of the territory in the overall mineral production. It should be noted that this measure remains a crude proxy, as we lack detailed information about mineral output. **Table I** in S5 Appendix presents summary statistics for the share of 3T and gold mines across treated and non-treated territories. **Table J** in S5 Appendix presents the regression results, which are in line with the main results.

Finally, we follow PV and conduct a robustness check in which we also control for territory-specific linear time trends. These allow us to control for the possibility that conflict events in a territory were already trending up or down before the introduction of DF. The results are presented in **Table K** in S5 Appendix and are in line with the main results.

## Conclusion

The US conflict minerals legislation, embodied by Section 1502 of the Dodd Frank Act, was passed by the US Congress in July 2010. Looking at the short-term impact (2011–2012) of Dodd-Frank, Parker and Vadheim [22] conclude that the legislation created an increase in violence, since armed groups compensated their income losses from taxing 3T mines by roving the countryside and increasing battles over gold mines.

We build on a much larger dataset of mining sites and extend the time horizon of the analysis by three years (2013–2015), thus covering the period 2004–2015. Our results echo the findings of Parker and Vadheim and confirm that Section 1502 does not do what it was intended to do. Like PV, we find that, in the short-term, the legislation strongly and significantly increased the likelihood of violent conflict in affected territories, especially in relatively unregulated gold mining areas. Battles between armed actors became more frequent, and events of looting and violence committed against civilians increased. In addition, we find that DF-targeted mining areas further witnessed a strong increase in riots, which is a clear sign of social upheaval. In the longer term, these effects seem to abate for the average DF-territory, while remaining highly significant for gold mining areas, which may suggest that rebels continue to fight for the control over gold sites. On a less pessimistic note, we do not find evidence that conflict events increased with the number of 3T mines in a territory. Moreover, when looking at the longer term, we find an indication that looting of civilians (slightly) decreased in 3T mining areas compared to the pre-DF period. This does not necessarily mean that the average civilian in this area has become less exposed to looting. DF has triggered a movement from 3T to gold mines, not only of armed actors, but also of artisanal miners. Hence, ‘per capita looting’ in 3T mining areas may not have decreased.

Our findings offer empirical support for recent studies–and an open letter signed by 70 academics and experts–that cast doubt on the ‘conflict minerals’ narrative of the Dodd-Frank legislation and other resource governance interventions [8,75]. More generally, they offer a cautionary tale about the potential unintended consequences of well-meaning international interventions that are based on strong assumptions of how natural resources relate to conflict.

Whether as part of a hard or soft law, conflict minerals awareness will likely not abate. Cuvelier et al. [15] describe how Section 1502 has functioned as a ‘wake-up’ call, in the sense that it has raised overall awareness–among Congolese civil society, electronics manufacturing companies, and governments worldwide–about the need of due diligence along the mineral supply chain. As such, it has resulted in the creation of a number of Congolese monitoring mechanisms and institutions to ensure a secure and transparent mining environment. Furthermore, the commotion around Section 1502 has sped up and served as a model (both of how to and how not to) for a European conflict minerals law, and even for Chinese Due Diligence Guidelines for Responsible Mineral Supply Chains [76,77]. Some have therefore argued that the suspension of Section 1502 will not have a big impact, because the risk of naming and shaming will continue exerting pressure on companies to keep their supply chains conflict free–whether supported by a formal law or not [78]. Others are more skeptical, highlighting that the suspension of Section 1502 removed the legal pressure to report on due diligence for tens of thousands of US companies, while the EU conflict minerals law only legally requires such reporting efforts for companies that exceed certain import thresholds [77].

On June 8, 2017, the US House of Representatives passed the Financial CHOICE Act. It aims to reverse many of Dodd-Frank’s provisions, and proposes to entirely repeal Section 1502 [79]. To come in effect, however, the Act must be passed by the Senate as well, which is unlikely according to many observers [80–82]. As of yet, no consequence has been given by US legislators to the statement made in President Trump’s executive order that “*a more effective means*” should be sought for “*breaking the link between commodities and armed groups in the DRC and adjoining countries*”. As clearly demonstrated by the local backfiring of Section 1502, such effective means are not straightforward. Any policy measure, targeting DRC or another war-torn region, will lack ambition if it keeps its narrow focus on the mineral supply chain, instead of taking a more systemic approach that seeks to address the root causes of the ongoing conflict. And, at the very least, the policy should give much more attention to so-called ‘accompanying measures’ that could strengthen mining communities, and mitigate unintended economic and political effects, including the effect on the short- and long(er)-run behavior of armed groups [77]. To anticipate these effects, consultation with local stakeholders is needed. Additionally, one could rely on the growing body of studies from scholars who gather first-hand data in war- and post-war areas.

## Supporting information

S1 Appendix Additional information on mining sites. (DOCX)

S2 Appendix Transmission of international to local mineral prices. (DOCX)

S3 Appendix Dynamic and spatial conflict lags. (DOCX)

S4 Appendix Main results in tabular form. (DOCX)

S5 Appendix Robustness checks. (DOCX)
