# Supplementary material for: More legislation, more violence? The impact of Dodd-Frank in the DRC
Source: PLoS One. 2018 Aug 9;13(8):e0201783. doi: 10.1371/journal.pone.0201783 (PMC6084930; doi:10.1371/journal.pone.0201783)
Supplement: S1 Appendix — (DOCX) [file pone.0201783.s001.docx]

# **S1 Appendix: Additional information on mining sites**

Information on mining sites comes from the International Peace Information Service (IPIS). PV make use of a database on the location of 659 artisanal mining sites, which was collected between 2008-2010, and was available on the IPIS website in June 2012. The IPIS dataset has since been significantly updated. New data collections took place in 2013-2014 and 2015 in which IPIS partnered up with the Congolese Ministry of Mines, other Congolese mining services and representatives from local civil society organizations.^[[1]](#footnote-1)^ The data and collection process of the different rounds are described in detail in various IPIS reports [1–3]. The latest update contains information on the location of 2,026 artisanal mining sites. There is some overlap between both databases. The combined dataset more than triples the number of artisanal mining sites compared to PV, to 2,282.

We illustrate the location of the artisanal mining sites in the PV study, and the combined databases in Fig A below; Table 1 in the paper shows summary statistics of both samples. In the PV set-up, the average territory contains 5.79 gold mines, 2.91 cassiterite mines, 0.31 coltan (tantalum) mines and 0.11 wolframite (tungsten) mines. Using the full database, the average territory contains 22.93 gold mines, 7.43 cassiterite mines, 1.80 coltan (tantalum) mines and 0.44 wolframite (tungsten) mines.

**Fig A. Artisanal mining sites**

| (a) PV set-up | (b) Full database |
| --- | --- |
| 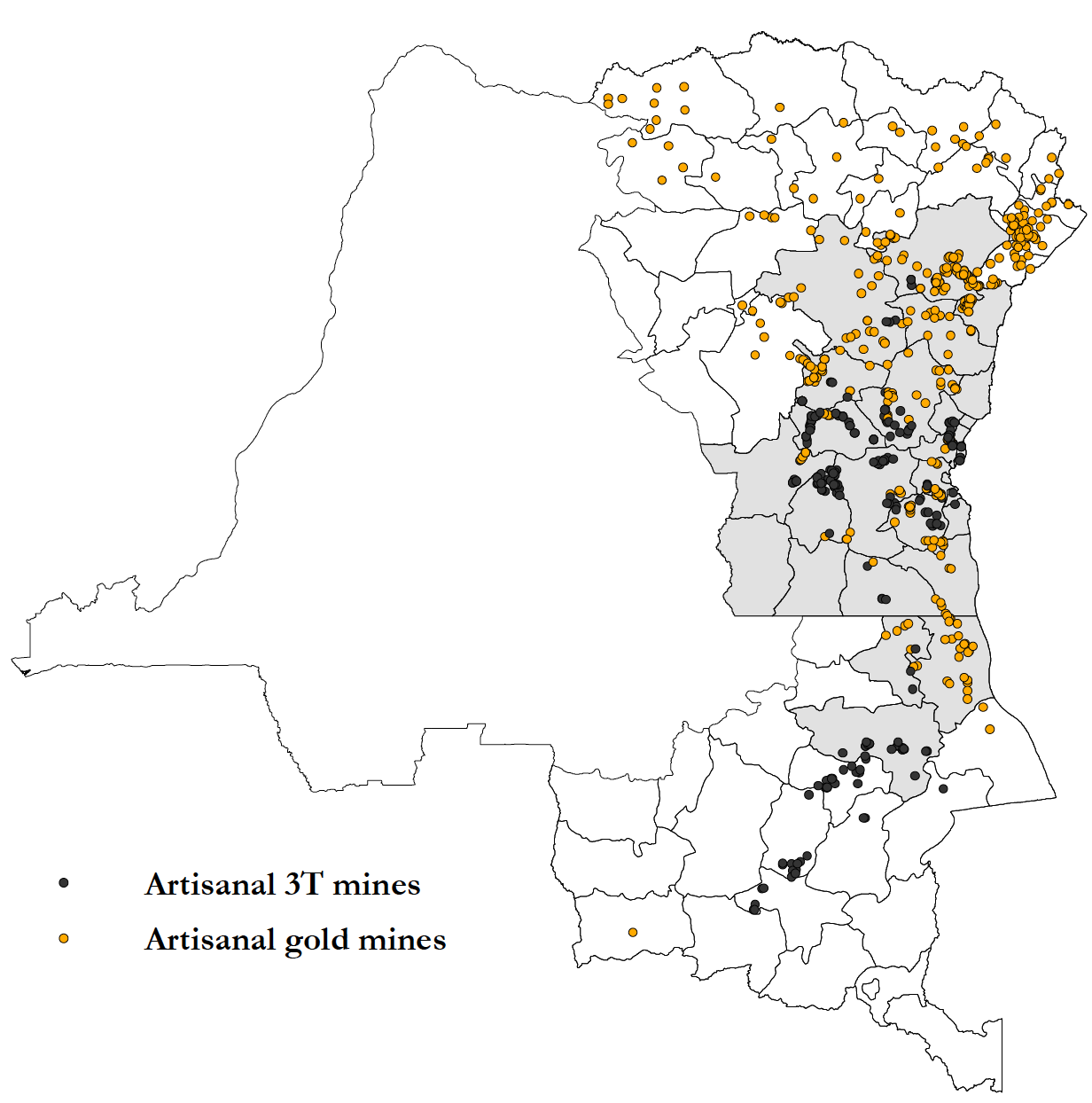 | **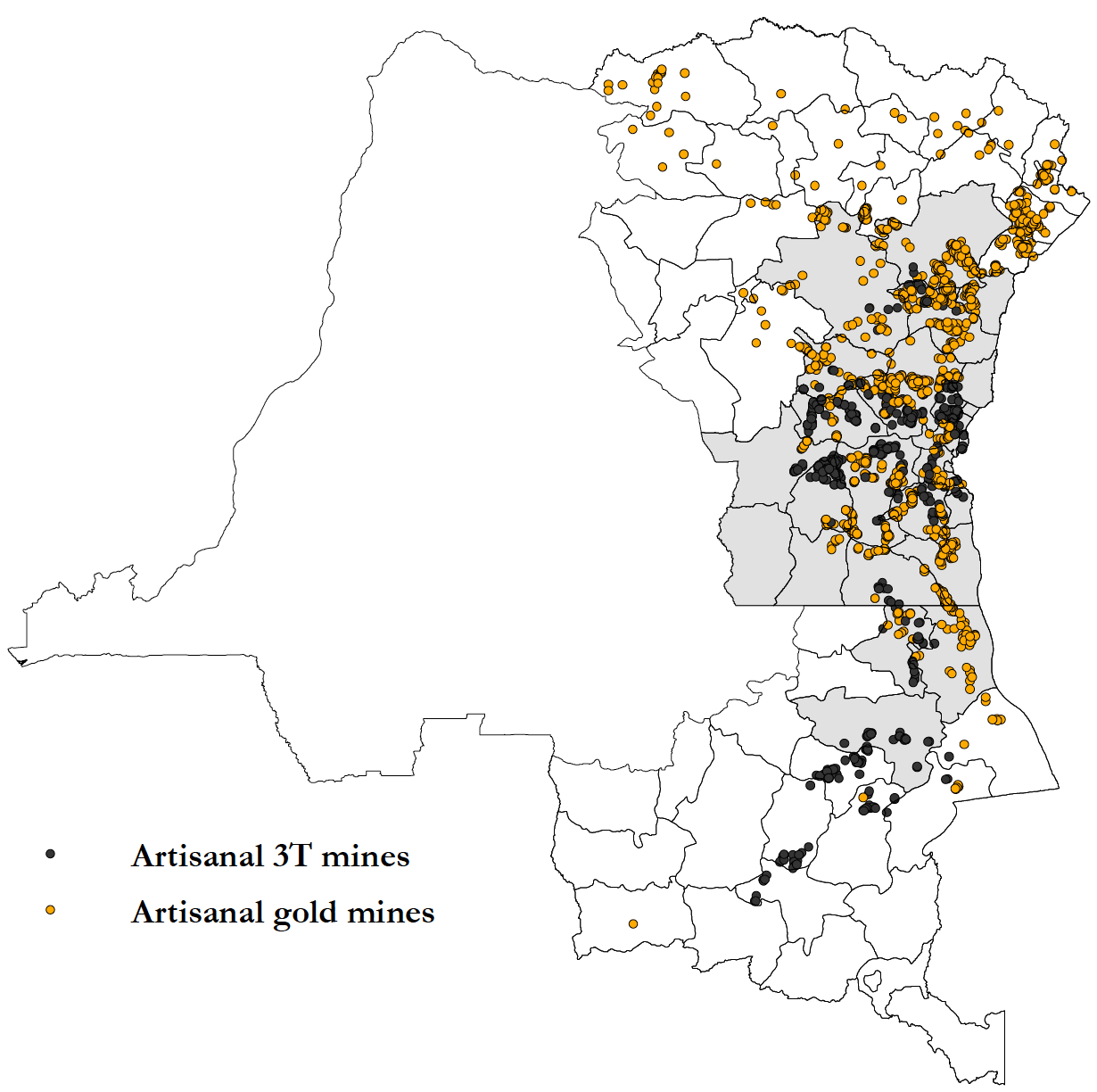** |

**Notes:** Panel (a) shows the location of artisanal 3T and gold mines as used in PV. Panel (b) shows the location of artisanal 3T and gold mines when combining the PV mining sites with the latest IPIS update. Shaded territories constitute the Dodd-Frank treatment area.

The increase in mining sites reflects the expanded geographic coverage of the mapping exercise rather than an increase in mining activities [1]. Based on the available information, it is likely that all mines in the full database were established before 2004 and existed throughout the entire period of study. First, while the opening date of mining sites is not recorded by IPIS, they believe that the large majority of mines existed before 2004 (personal communication with IPIS). The sites included in the update of the database had not been visited prior to 2015 for logistical and security reasons [1]. As such, the PV sample may have been biased towards more accessible mining sites, which are more likely to be affected by legislation compared to mines operating ‘under the radar’. The updated IPIS dataset thus calls for a replication of the PV study. Second, by way of corroboration, we turn to the analysis of Sanchez de la Sierra [4]. He collected data on 411 artisanal mining sites in North- and South-Kivu. In North-Kivu he sampled all communities with mining activities, while in South-Kivu he sampled all coltan-mining communities and a random subset of the gold-mining communities. Of the 411 artisanal mining sites in his sample, all but one site existed already before 1995. Moreover, the minerals in none of these sites were exhausted before the end of his study, in 2013.

Detailed information on the evolution of mining output is not available for the mining sites in the IPIS database. Hence, we use a time-invariant measure of 3T or gold endowment. In doing so, we follow the example of PV [5] and Sanchez de la Sierra [4]. As indicated by Sanchez de la Sierra, this approach has the advantage that it allows us to circumvent the fact that mining output is endogenous to mineral prices and conflict.

**References**

[1] Weyns Y, Hoex L, Matthysen K. Analysis of the Interactive Map of Artisanal Mining Areas in Eastern DR Congo. 2015 Update. Antwerp: IPIS; 2016.

[2] Spittaels S, Hilgert F. Analysis of the Interactive Map of Artisanal Mining Areas in Eastern DR Congo. Antwerp: IPIS; 2013.

[3] Spittaels S, Matthysen K, Weyns Y, Hilgert F, Bulzomi A. Analysis of the Interactive Map of Artisanal Mining Areas in Eastern DR Congo: May 2014 Update. Antwerp: IPIS; 2014.

[4] Sanchez de la Sierra R. On the Origins of the State: Stationary Bandits and Taxation in Eastern Congo 2017.

[5] Parker DP, Vadheim B. Resource Cursed or Policy Cursed? US Regulation of Conflict Minerals and Violence in the Congo. Journal of the Association of Environmental and Resource Economists 2017; 4:1–49.

1. Other local stakeholders include: the Congolese Mining Registry (CAMI); the Congolese Public Service for Assistance to Artisanal- and Small-scale Mining (SAESSCAM), the provincial Mining Divisions and local civil society organizations. The data collection and analysis were funded by the World Bank’s PROMINES program, the International Organization for Migration and the Belgian Ministry of Foreign Affairs [1]. [↑](#footnote-ref-1)
