# Supplementary material for: More legislation, more violence? The impact of Dodd-Frank in the DRC
Source: PLoS One. 2018 Aug 9;13(8):e0201783. doi: 10.1371/journal.pone.0201783 (PMC6084930; doi:10.1371/journal.pone.0201783)
Supplement: S2 Appendix — (DOCX) [file pone.0201783.s002.docx]

# **S2 Appendix: Transmission of international to local mineral prices**

Following the example of Parker & Vadheim [1], the analysis makes use of international mineral prices. We do not have detailed information on local mineral prices. Fieldwork by Geenen [2] indicates, however, that local mineral traders in Eastern Congo closely monitor world mineral prices and use them to set local prices. This also happens at the very local level: “Even small traders who are based near the mining sites say they regularly check the price online, on their phone, or on TV5 Afrique” [2: p.249]. Geenen further quotes a local mineral trader stating that “Following the world market price is the least we can do. If you don’t do it, you lose money” [2: p.249].

The transmission from international to local prices may however have been distorted after the introduction of the Dodd-Frank act. Although artisanal mining communities were affected by the de-facto embargo^[[1]](#footnote-1)^, mineral trade did not stop entirely. First, minerals were smuggled across the DRC’s eastern borders [8]. This was especially the case for gold, which is easy to conceal and for which most of the production was already smuggled out of the country before the introduction of Dodd-Frank [9,10]. Second, Chinese buyers, who were not affected by the Dodd-Frank act, continued to export 3T minerals from the DRC [8]. Research by the Southern Africa Research Watch indicates that buyers took advantage of the situation to buy minerals at heavily discounted prices from artisanal miners [11].

We are not particularly interested in the estimated coefficients for the mineral price variables, but rather control for them to test the robustness of our β coefficients. Controlling for international price variables rather than local prices has the advantage that they are much less likely to be endogenous to the local context; i.e. they are much less likely to be affected by the local conflict situation or fluctuations in local mineral production. For instance, the large majority of mining sites in our sample (72.3%) are gold mining sites, and the DRC supplies less than 1% of world gold production [12]. And while an estimated 10-20% of the world production of tantalum originates from the DRC [12,13], coltan (tantalum) mines only comprise 5.5% or the mining sites in our sample. The results are further robust to dropping the tantalum price x mines interactions from the analysis (results not reported but available from the authors upon request).

**References**

[1] Parker DP, Vadheim B. Resource Cursed or Policy Cursed? US Regulation of Conflict Minerals and Violence in the Congo. Journal of the Association of Environmental and Resource Economists 2017;4:1–49.

[2] Geenen S. “Qui Cherche, Trouve” the Political Economy of Access to Gold Mining and Trade in South Kivu, DRC 2014.

[3] Cuvelier J, Van Bockstael S, Vlassenroot K, Iguma C. Analyzing the Impact of the Dodd-Frank Act on Congolese Livelihoods. SSRC Conflict Prevention and Peace Forum 2014.

[4] Geenen S. A dangerous Bet: The Challenges of Formalizing Artisanal Mining in DRC. Resources Policy 2012;37:322–30. doi:10.1016/j.resourpol.2012.02.004.

[5] Seay L. What’s Wrong with Dodd-Frank 1502? Conflict Minerals, Civilian Livelihoods, and the Unintended Consequences of Western Advocacy. Center for Global Development; 2012.

[6] Wimmer SZ, Hilgert F. Bisie. A One-Year Snapshot of the DRC’s Principal Cassiterite Mine. International Peace Information Service; 2011.

[7] Parker DP, Foltz JD, Elsea D. Unintended Consequences of Economic Sanctions for Human Rights. Conflict Minerals and Infant Mortality in the Democratic Republic of the Congo. UNU-WIDER; 2016.

[8] UN security council. Letter Dated 29 November 2011 from the Chair of the Security Council Committee Established Pursuant to Resolution 1533 (2004) Concerning the Democratic Republic of the Congo Addressed to the President of the Security Council. United Nations Security Council; 2011.

[9] De Koning R. Conflict Minerals in the Democratic Republic of the Congo: Aligning Trade and Security Interventions. Solna, Sweden: Stockholm International Peace Research Institute; 2011.

[10] World Bank. Democratic Republic of Congo - Growth with Governance in the Mining Sector. World Bank; 2008.

[11] Carisch E. Conflict Gold to Criminal Gold: The New Face of Artisanal Gold Mining in Congo. Southern Africa Resource Watch; 2012.

[12] The Enough Project. A Comprehensive Approach to Congo’s Conflict Minerals. The Enough Project; 2009.

[13] USGS. Mineral Commodity Summaries, 2013. United States Geological Survey; 2013.

1. Qualitative evidence suggests that people in mining communities could no longer afford to visit healthcare facilities or pay for their children’s schooling; moreover, the economic effects where felt throughout the eastern provinces as artisanal miners could no longer afford to pay for goods, services and agricultural products [3–6]. Using quantitative data, Parker et al. [7] further find that the probability of infant deaths increased by at least 143% in villages near artisanal mines targeted by the Dodd-Frank act. [↑](#footnote-ref-1)
