# Supplementary material for: More legislation, more violence? The impact of Dodd-Frank in the DRC
Source: PLoS One. 2018 Aug 9;13(8):e0201783. doi: 10.1371/journal.pone.0201783 (PMC6084930; doi:10.1371/journal.pone.0201783)
Supplement: S3 Appendix — (DOCX) [file pone.0201783.s003.docx]

# **S3 Appendix: Dynamic and spatial conflict lags**

Following the example of Parker & Vadheim [1], we include lags for the incidence of conflict events in previous months ($\mathrm{conflict}_{i,t-x}$) and in adjacent territories ($\mathrm{adj}.\mathrm{conflict}_{i,t-x}$). These dynamic and spatial lags capture the incidence of all types of conflict events, thus taking into account that past battles may affect e.g. future violence against civilians and looting, or the other way around. Indeed, we find a high and significant correlation between the four conflict indicators (see Table A).

**Table A. Correlation between conflict indicators**

|  | Looting | Battles | VAC | Riots |
| --- | --- | --- | --- | --- |
| Looting | 1.00 |  |  |  |
|  |  |  |  |  |
| Battles | 0.62 | 1.00 |  |  |
|  | (0.00) |  |  |  |
| VAC | 0.86 | 0.69 | 1.00 |  |
|  | (0.00) | (0.00) |  |  |
| Riots | 0.55 | 0.42 | 0.48 | 1.00 |
|  | (0.00) | (0.00) | (0.00) |  |
| **Notes:** This Table presents the tetrachoric correlation coefficients for the four conflict event indicators. P-values are reported in parentheses. VAC stands for Violence against civilians. | | | | |

We realize that the coefficients on the dynamic and spatial conflict lags may be estimated with bias. The introduction of lagged conflict variables gives rise to ‘dynamic panel bias’, i.e. the lags are correlated with the error term [2]. Since we perform within-cell estimations, our estimates of the lags would understate the actual persistence of conflict. However, the bias is likely to be small since it decreases with the number of time periods, which is large in our case; i.e. 144 months [3]. Introducing spatial lags gives rise to a simultaneity or reflection problem, since it is unclear if conflict in a specific cell is driven by conflict in adjacent cells, or the other way around [4,5]. A positive correlation of conflict across adjacent cells would overstate the estimated coefficients on the spatial conflict lags. The coefficients on spatial lags may thus be estimated with bias, but we only introduce them to check the robustness of our β coefficients.

When presenting the main results, we follow Parker & Vadheim [1] and include 3-month lags for within-territory conflict (*x* ∈{0,3}), as well as contemporaneous conflict in adjacent territories and a 1-month lag (*x* ∈{0,1}). The results are however robust to adding up to 12-month dynamic and spatial lags (see the section ‘Robustness’).

**References**

[1] Parker DP, Vadheim B. Resource Cursed or Policy Cursed? US Regulation of Conflict Minerals and Violence in the Congo. Journal of the Association of Environmental and Resource Economists 2017; 4:1–49.

[2] Nickell S. Biases in Dynamic Models with Fixed Effects. Econometrica 1981; 49:1417–26.

[3] Roodman D. How to do xtabond2: An introduction to difference and system GMM in Stata. The Stata Journal 2009; 9:86–136.

[4] Anselin L. Under the Hood Issues in the Specification and Interpretation of Spatial Regression Models. Agricultural Economics 2002; 27:247–67.

[5] Manski CF. Identification of Endogenous Social Effects: The Reflection Problem. The Review of Economic Studies 1993; 60:531–42.
