# Supplementary material for: More legislation, more violence? The impact of Dodd-Frank in the DRC
Source: PLoS One. 2018 Aug 9;13(8):e0201783. doi: 10.1371/journal.pone.0201783 (PMC6084930; doi:10.1371/journal.pone.0201783)
Supplement: S4 Appendix — (DOCX) [file pone.0201783.s004.docx]

# **S4 Appendix: Main results in tabular form**

This appendix presents the full set of results in tabular form. Tables B, C and D – presented on the following pages – correspond to Panels (a), (b) and (c) of Figure 4 in the paper. While the Panels in Figure 4 only present results from the most inclusive specifications, the below Tables present the results from several model specifications, going from parsimonious to more inclusive.

**Table B. PV set-up**

|  | Looting indicator | | | Battles indicator | | | Violence indicator | | | Riots indicator | | |
| --- | --- | --- | --- | --- | --- | --- | --- | --- | --- | --- | --- | --- |
|  | (1) | (2) | (3) | (4) | (5) | (6) | (7) | (8) | (9) | (10) | (11) | (12) |
| DF | 0.057^**^ | 0.055^**^ | 0.047^**^ | 0.081^***^ | 0.073^**^ | 0.043^*^ | 0.088^***^ | 0.099^***^ | 0.074^***^ | 0.050^***^ | 0.048^***^ | 0.045^***^ |
|  | (0.023) | (0.024) | (0.022) | (0.030) | (0.033) | (0.026) | (0.032) | (0.034) | (0.027) | (0.017) | (0.017) | (0.017) |
| DF * 3T mines | -0.002 | -0.003 | -0.003 | 0.007 | 0.003 | -0.000 | -0.010 | -0.002 | -0.004 | -0.014 | -0.016 | -0.016 |
|  | (0.015) | (0.015) | (0.014) | (0.028) | (0.025) | (0.021) | (0.026) | (0.028) | (0.024) | (0.010) | (0.011) | (0.011) |
| DF * gold mines | 0.011 | 0.006 | 0.003 | 0.042^***^ | 0.045^***^ | 0.038^***^ | 0.029^*^ | 0.020 | 0.013 | -0.003 | -0.005 | -0.006 |
|  | (0.011) | (0.010) | (0.008) | (0.015) | (0.015) | (0.011) | (0.017) | (0.015) | (0.012) | (0.008) | (0.007) | (0.007) |
|  |  |  |  |  |  |  |  |  |  |  |  |  |
| Gold p * gold indicator |  | 0.013^**^ | 0.013^**^ |  | -0.000 | -0.002 |  | 0.019^*^ | 0.018^**^ |  | 0.007 | 0.007 |
| Tin p * cassiterite indicator |  | -0.002 | -0.001 |  | -0.001 | 0.002 |  | -0.026 | -0.025 |  | -0.011^*^ | -0.011^*^ |
| Tantalum p * coltan indicator |  | -0.000 | 0.000 |  | -0.009 | -0.000 |  | -0.001 | 0.007 |  | -0.001 | -0.001 |
| Tungsten p * wolframite indicator |  | -0.004 | -0.004 |  | 0.017 | 0.016 |  | -0.005 | -0.005 |  | 0.014 | 0.014 |
|  |  |  |  |  |  |  |  |  |  |  |  |  |
| Wet season indicator |  | -0.001 | -0.001 |  | 0.003 | 0.002 |  | 0.008 | 0.007 |  | -0.005 | -0.005 |
| Dry season indicator |  | 0.005 | 0.006 |  | -0.003 | 0.000 |  | 0.004 | 0.007 |  | -0.001 | -0.001 |
| Rainfall anomalies |  | 0.002 | 0.002 |  | -0.001 | 0.000 |  | 0.003 | 0.004 |  | 0.003 | 0.003 |
| 1 month lag rainfall anomalies |  | -0.001 | -0.000 |  | -0.004 | -0.004 |  | -0.002 | -0.002 |  | -0.002 | -0.002 |
| 2 month lag rainfall anomalies |  | 0.004 | 0.005^*^ |  | 0.000 | 0.001 |  | -0.009^*^ | -0.008^*^ |  | 0.000 | 0.000 |
| Rainfall anomalies2 |  | -0.005^**^ | -0.005^*^ |  | -0.005^**^ | -0.004^*^ |  | -0.005 | -0.004 |  | -0.000 | -0.001 |
| 1 month lag rainfall anomalies2 |  | -0.002 | -0.002 |  | 0.000 | 0.001 |  | -0.002 | -0.002 |  | -0.002^*^ | -0.002^*^ |
| 2 month lag rainfall anomalies2 |  | -0.002 | -0.002 |  | 0.010^***^ | 0.009^***^ |  | -0.000 | -0.001 |  | -0.002^*^ | -0.002^**^ |
|  |  |  |  |  |  |  |  |  |  |  |  |  |
| 1 month lagged conflict |  |  | 0.022^*^ |  |  | 0.113^***^ |  |  | 0.096^***^ |  |  | 0.014 |
| 2 month lagged conflict |  |  | 0.037^**^ |  |  | 0.079^***^ |  |  | 0.059^***^ |  |  | -0.005 |
| 3 month lagged conflict |  |  | 0.018 |  |  | 0.042^**^ |  |  | 0.048^**^ |  |  | 0.016^*^ |
| Adjacent territory conflict |  |  | 0.005 |  |  | 0.036^***^ |  |  | 0.022^**^ |  |  | 0.011^*^ |
| 1 month lagged adj. territory conflict |  |  | 0.002 |  |  | 0.020^*^ |  |  | 0.019 |  |  | -0.007^*^ |
|  |  |  |  |  |  |  |  |  |  |  |  |  |
| Territory FE | Yes | Yes | Yes | Yes | Yes | Yes | Yes | Yes | Yes | Yes | Yes | Yes |
| Month FE | Yes | Yes | Yes | Yes | Yes | Yes | Yes | Yes | Yes | Yes | Yes | Yes |
| Observations | 7,560 | 7,420 | 7,350 | 7,560 | 7,420 | 7,350 | 7,560 | 7,420 | 7,350 | 7,560 | 7,420 | 7,350 |
| **Notes:** *** p<0.01, ** p<0.05, * p<0.1; All specifications are estimated using a Linear Probability Model; Conley (1999) standard errors in parentheses, allowing for spatial correlation within a 500 km radius and for infinite serial correlation; Results in this Table are based on the PV set-up: smaller sample of mines, short time horizon (2004-2012). | | | | | | | | | | | | |

**Table C. More mines**

|  | Looting indicator | | | Battles indicator | | | Violence indicator | | | Riots indicator | | |
| --- | --- | --- | --- | --- | --- | --- | --- | --- | --- | --- | --- | --- |
|  | (1) | (2) | (3) | (4) | (5) | (6) | (7) | (8) | (9) | (10) | (11) | (12) |
| DF | 0.039^**^ | 0.039^**^ | 0.033^**^ | 0.063^**^ | 0.073^*^ | 0.053^**^ | 0.057^*^ | 0.082^**^ | 0.062^**^ | 0.044^**^ | 0.039^**^ | 0.036^**^ |
|  | (0.018) | (0.019) | (0.016) | (0.030) | (0.038) | (0.027) | (0.031) | (0.037) | (0.029) | (0.017) | (0.019) | (0.017) |
| DF * 3T mines | -0.002 | -0.004 | -0.004 | -0.002 | -0.001 | -0.001 | -0.001 | 0.006 | 0.006 | -0.000 | -0.002 | -0.003 |
|  | (0.005) | (0.005) | (0.005) | (0.011) | (0.008) | (0.006) | (0.012) | (0.011) | (0.010) | (0.006) | (0.005) | (0.005) |
| DF * gold mines | 0.007^***^ | 0.006^***^ | 0.005^***^ | 0.013^***^ | 0.014^***^ | 0.011^***^ | 0.016^***^ | 0.015^***^ | 0.012^***^ | -0.001 | -0.002 | -0.003 |
|  | (0.002) | (0.001) | (0.001) | (0.003) | (0.003) | (0.003) | (0.005) | (0.005) | (0.004) | (0.002) | (0.002) | (0.002) |
|  |  |  |  |  |  |  |  |  |  |  |  |  |
| Gold p * gold indicator |  | 0.001 | 0.001 |  | -0.009 | -0.009 |  | 0.014 | 0.014^*^ |  | 0.006 | 0.006 |
| Tin p * cassiterite indicator |  | -0.019^*^ | -0.017^*^ |  | -0.022 | -0.017 |  | -0.036^*^ | -0.031^*^ |  | -0.018^**^ | -0.018^**^ |
| Tantalum p * coltan indicator |  | 0.016^**^ | 0.015^**^ |  | 0.012 | 0.006 |  | -0.005 | -0.008 |  | 0.009^*^ | 0.009^*^ |
| Tungsten p * wolframite indicator |  | 0.005 | 0.005 |  | 0.000 | -0.000 |  | -0.009 | -0.010 |  | 0.017 | 0.017 |
|  |  |  |  |  |  |  |  |  |  |  |  |  |
| Wet season indicator |  | -0.007 | -0.006 |  | 0.004 | 0.004 |  | 0.004 | 0.004 |  | 0.001 | 0.001 |
| Dry season indicator |  | 0.002 | 0.003 |  | -0.005 | -0.002 |  | -0.001 | 0.002 |  | 0.002 | 0.002 |
| Rainfall anomalies |  | -0.001 | -0.000 |  | -0.002 | -0.000 |  | 0.002 | 0.003 |  | 0.003 | 0.003 |
| 1 month lag rainfall anomalies |  | -0.000 | -0.000 |  | -0.005 | -0.005 |  | -0.002 | -0.002 |  | -0.002 | -0.002 |
| 2 month lag rainfall anomalies |  | 0.002 | 0.002 |  | -0.001 | 0.000 |  | -0.010^*^ | -0.009^*^ |  | -0.000 | 0.000 |
| Rainfall anomalies2 |  | -0.005^**^ | -0.004^*^ |  | -0.003 | -0.002 |  | -0.005 | -0.004 |  | -0.001 | -0.001 |
| 1 month lag rainfall anomalies2 |  | -0.002 | -0.001 |  | 0.001 | 0.002 |  | -0.002 | -0.002 |  | -0.002^*^ | -0.002^*^ |
| 2 month lag rainfall anomalies2 |  | -0.001 | -0.001 |  | 0.011^***^ | 0.010^***^ |  | -0.000 | -0.001 |  | -0.002^*^ | -0.002^**^ |
|  |  |  |  |  |  |  |  |  |  |  |  |  |
| 1 month lagged conflict |  |  | 0.028^**^ |  |  | 0.112^***^ |  |  | 0.092^***^ |  |  | 0.015 |
| 2 month lagged conflict |  |  | 0.029^**^ |  |  | 0.079^***^ |  |  | 0.055^***^ |  |  | -0.005 |
| 3 month lagged conflict |  |  | 0.009 |  |  | 0.038^*^ |  |  | 0.044^**^ |  |  | 0.016^*^ |
| Adjacent territory conflict |  |  | 0.003 |  |  | 0.008 |  |  | 0.022^***^ |  |  | 0.010^*^ |
| 1 month lagged adj. territory conflict |  |  | 0.007 |  |  | 0.017 |  |  | 0.019 |  |  | -0.007^*^ |
|  |  |  |  |  |  |  |  |  |  |  |  |  |
| Territory FE | Yes | Yes | Yes | Yes | Yes | Yes | Yes | Yes | Yes | Yes | Yes | Yes |
| Month FE | Yes | Yes | Yes | Yes | Yes | Yes | Yes | Yes | Yes | Yes | Yes | Yes |
| Observations | 7,560 | 7,420 | 7,350 | 7,560 | 7,420 | 7,350 | 7,560 | 7,420 | 7,350 | 7,560 | 7,420 | 7,350 |
| **Notes:** *** p<0.01, ** p<0.05, * p<0.1; All specifications are estimated using a Linear Probability Model; Conley (1999) standard errors in parentheses, allowing for spatial correlation within a 500 km radius and for infinite serial correlation; Results in this Table are based on the full sample of mines, for the period 2004-2012. | | | | | | | | | | | | |

**Table D. More mines & Longer time horizon**

|  | Looting indicator | | | Battles indicator | | | Violence indicator | | | Riots indicator | | |
| --- | --- | --- | --- | --- | --- | --- | --- | --- | --- | --- | --- | --- |
|  | (1) | (2) | (3) | (4) | (5) | (6) | (7) | (8) | (9) | (10) | (11) | (12) |
| DF | 0.022 | 0.018 | 0.012 | 0.075^**^ | 0.076^*^ | 0.058^*^ | 0.040 | 0.039 | 0.021 | 0.039^*^ | 0.043^*^ | 0.042^*^ |
|  | (0.015) | (0.017) | (0.015) | (0.036) | (0.043) | (0.031) | (0.034) | (0.041) | (0.034) | (0.021) | (0.022) | (0.022) |
| DF * 3T mines | -0.003 | -0.005 | -0.005^*^ | 0.008 | 0.007 | 0.006 | 0.003 | 0.003 | 0.002 | -0.001 | -0.000 | -0.001 |
|  | (0.004) | (0.003) | (0.003) | (0.010) | (0.008) | (0.006) | (0.009) | (0.008) | (0.008) | (0.007) | (0.007) | (0.007) |
| DF * gold mines | 0.006^***^ | 0.005^***^ | 0.004^***^ | 0.015^***^ | 0.016^***^ | 0.012^***^ | 0.012^***^ | 0.010^***^ | 0.007^**^ | 0.001 | 0.000 | -0.000 |
|  | (0.001) | (0.001) | (0.001) | (0.003) | (0.003) | (0.002) | (0.003) | (0.003) | (0.003) | (0.002) | (0.002) | (0.002) |
|  |  |  |  |  |  |  |  |  |  |  |  |  |
| Gold p * gold indicator |  | 0.004 | 0.005 |  | -0.016 | -0.015^*^ |  | 0.018^*^ | 0.019^**^ |  | 0.005 | 0.006 |
| Tin p * cassiterite indicator |  | -0.015 | -0.014 |  | -0.017 | -0.013 |  | -0.022 | -0.019 |  | -0.023^**^ | -0.022^**^ |
| Tantalum p * coltan indicator |  | 0.014^**^ | 0.013^**^ |  | 0.014 | 0.008 |  | -0.001 | -0.004 |  | 0.003 | 0.003 |
| Tungsten p * wolframite indicator |  | 0.011 | 0.009 |  | 0.004 | 0.001 |  | 0.015 | 0.011 |  | 0.007 | 0.008 |
|  |  |  |  |  |  |  |  |  |  |  |  |  |
| Wet season indicator |  | -0.006 | -0.006 |  | -0.000 | -0.001 |  | 0.003 | 0.003 |  | 0.007 | 0.007 |
| Dry season indicator |  | 0.001 | 0.001 |  | -0.005 | -0.004 |  | 0.006 | 0.007 |  | 0.006 | 0.006 |
| Rainfall anomalies |  | -0.003 | -0.003 |  | -0.000 | 0.001 |  | 0.002 | 0.003 |  | -0.000 | -0.000 |
| 1 month lag rainfall anomalies |  | -0.001 | -0.001 |  | -0.003 | -0.003 |  | -0.001 | -0.001 |  | -0.001 | -0.001 |
| 2 month lag rainfall anomalies |  | 0.001 | 0.001 |  | -0.000 | 0.000 |  | -0.006 | -0.006 |  | -0.003 | -0.003 |
| Rainfall anomalies2 |  | -0.002 | -0.002 |  | -0.002 | -0.002 |  | -0.003 | -0.003 |  | 0.002 | 0.002 |
| 1 month lag rainfall anomalies2 |  | -0.000 | -0.000 |  | 0.002 | 0.001 |  | -0.002 | -0.002 |  | -0.000 | -0.000 |
| 2 month lag rainfall anomalies2 |  | 0.000 | 0.000 |  | 0.008^***^ | 0.008^***^ |  | 0.001 | 0.001 |  | -0.000 | -0.000 |
|  |  |  |  |  |  |  |  |  |  |  |  |  |
| 1 month lagged conflict |  |  | 0.023^***^ |  |  | 0.113^***^ |  |  | 0.089^***^ |  |  | 0.020^**^ |
| 2 month lagged conflict |  |  | 0.033^***^ |  |  | 0.080^***^ |  |  | 0.054^***^ |  |  | -0.001 |
| 3 month lagged conflict |  |  | 0.016 |  |  | 0.052^***^ |  |  | 0.045^***^ |  |  | 0.006 |
| Adjacent territory conflict |  |  | -0.002 |  |  | -0.000 |  |  | 0.023^***^ |  |  | 0.007 |
| 1 month lagged adj. territory conflict |  |  | 0.009^*^ |  |  | 0.016 |  |  | 0.014 |  |  | -0.015^***^ |
|  |  |  |  |  |  |  |  |  |  |  |  |  |
| Territory FE | Yes | Yes | Yes | Yes | Yes | Yes | Yes | Yes | Yes | Yes | Yes | Yes |
| Month FE | Yes | Yes | Yes | Yes | Yes | Yes | Yes | Yes | Yes | Yes | Yes | Yes |
| Observations | 10,080 | 9,940 | 9,870 | 10,080 | 9,940 | 9,870 | 10,080 | 9,940 | 9,870 | 10,080 | 9,940 | 9,870 |
| **Notes:** *** p<0.01, ** p<0.05, * p<0.1; All specifications are estimated using a Linear Probability Model; Conley (1999) standard errors in parentheses, allowing for spatial correlation within a 500 km radius and for infinite serial correlation; Results in this Table are based on the full sample of mines for the longer time horizon (2004-2015). | | | | | | | | | | | | |
