# Supplementary material for: More legislation, more violence? The impact of Dodd-Frank in the DRC
Source: PLoS One. 2018 Aug 9;13(8):e0201783. doi: 10.1371/journal.pone.0201783 (PMC6084930; doi:10.1371/journal.pone.0201783)
Supplement: S5 Appendix — (DOCX) [file pone.0201783.s005.docx]

# **S5 Appendix: Robustness checks**

This appendix contains the results of the robustness checks described in the section ‘Robustness’.

**Table E. Standard errors**

| *Dependent variable:* | **Looting** | | | **Battles** | | | **Violence** | | | **Riots** | | |
| --- | --- | --- | --- | --- | --- | --- | --- | --- | --- | --- | --- | --- |
| *Variable of interest:* | DF | DF * 3T mines | DF * gold mines | DF | DF * 3T mines | DF * gold mines | DF | DF * 3T mines | DF * gold mines | DF | DF * 3T mines | DF * gold mines |
|  | (1) | (2) | (3) | (4) | (5) | (6) | (7) | (8) | (9) | (10) | (11) | (12) |
|  |  |  |  |  |  |  |  |  |  |  |  |  |
| **PV-set up** (7,350 obs.) | 0.047 | -0.003 | 0.003 | 0.043 | -0.000 | 0.038 | 0.074 | -0.004 | 0.013 | 0.045 | -0.016 | -0.006 |
| Spatial: 1,000 km. Time: infinite | (0.023) | (0.014) | (0.008) | (0.026) | (0.020) | (0.011) | (0.028) | (0.024) | (0.012) | (0.017) | (0.010) | (0.007) |
| Spatial: 100 km. Time: infinite | (0.021) | (0.015) | (0.009) | (0.025) | (0.022) | (0.011) | (0.027) | (0.025) | (0.012) | (0.018) | (0.011) | (0.007) |
| Spatial: 100 km. Time: 5 years | (0.017) | (0.012) | (0.008) | (0.021) | (0.018) | (0.010) | (0.024) | (0.020) | (0.012) | (0.016) | (0.009) | (0.005) |
| Spatial: 100 km. Time: 1 year | (0.016) | (0.010) | (0.008) | (0.021) | (0.016) | (0.010) | (0.023) | (0.017) | (0.015) | (0.015) | (0.007) | (0.005) |
| Territory-level | (0.021) | (0.015) | (0.009) | (0.026) | (0.022) | (0.011) | (0.028) | (0.025) | (0.012) | (0.018) | (0.011) | (0.007) |
|  |  |  |  |  |  |  |  |  |  |  |  |  |
| **More mines** (7,350 obs.) | 0.033 | -0.004 | 0.005 | 0.053 | -0.001 | 0.011 | 0.062 | 0.006 | 0.012 | 0.036 | -0.003 | -0.003 |
| Spatial: 1,000 km. Time: infinite | (0.017) | (0.004) | (0.001) | (0.027) | (0.006) | (0.003) | (0.029) | (0.010) | (0.004) | (0.017) | (0.005) | (0.002) |
| Spatial: 100 km. Time: infinite | (0.017) | (0.005) | (0.002) | (0.027) | (0.006) | (0.003) | (0.029) | (0.010) | (0.004) | (0.018) | (0.005) | (0.002) |
| Spatial: 100 km. Time: 5 years | (0.015) | (0.005) | (0.002) | (0.022) | (0.007) | (0.003) | (0.023) | (0.009) | (0.004) | (0.016) | (0.005) | (0.002) |
| Spatial: 100 km. Time: 1 year | (0.016) | (0.005) | (0.002) | (0.020) | (0.008) | (0.004) | (0.022) | (0.008) | (0.004) | (0.015) | (0.004) | (0.002) |
| Territory-level | (0.017) | (0.005) | (0.002) | (0.027) | (0.006) | (0.003) | (0.029) | (0.010) | (0.004) | (0.018) | (0.005) | (0.002) |
|  |  |  |  |  |  |  |  |  |  |  |  |  |
| **More mines & Longer time horizon** (9,870 obs.) | 0.012 | -0.005 | 0.004 | 0.058 | 0.006 | 0.012 | 0.021 | 0.002 | 0.007 | 0.042 | -0.001 | -0.000 |
| Spatial: 1,000 km. Time: infinite | (0.015) | (0.003) | (0.001) | (0.031) | (0.006) | (0.002) | (0.034) | (0.008) | (0.003) | (0.021) | (0.007) | (0.002) |
| Spatial: 100 km. Time: infinite | (0.015) | (0.003) | (0.001) | (0.031) | (0.006) | (0.002) | (0.034) | (0.008) | (0.003) | (0.022) | (0.007) | (0.002) |
| Spatial: 100 km. Time: 5 years | (0.012) | (0.003) | (0.001) | (0.021) | (0.006) | (0.003) | (0.024) | (0.006) | (0.003) | (0.015) | (0.005) | (0.002) |
| Spatial: 100 km. Time: 1 year | (0.012) | (0.004) | (0.002) | (0.017) | (0.006) | (0.003) | (0.020) | (0.006) | (0.003) | (0.013) | (0.004) | (0.001) |
| Territory-level | (0.015) | (0.003) | (0.001) | (0.031) | (0.006) | (0.002) | (0.034) | (0.008) | (0.003) | (0.022) | (0.007) | (0.002) |
|  |  |  |  |  |  |  |  |  |  |  |  |  |
| Territory FE | Yes | Yes | Yes | Yes | Yes | Yes | Yes | Yes | Yes | Yes | Yes | Yes |
| Month FE | Yes | Yes | Yes | Yes | Yes | Yes | Yes | Yes | Yes | Yes | Yes | Yes |
| Price controls | Yes | Yes | Yes | Yes | Yes | Yes | Yes | Yes | Yes | Yes | Yes | Yes |
| Weather controls | Yes | Yes | Yes | Yes | Yes | Yes | Yes | Yes | Yes | Yes | Yes | Yes |
| Conflict controls | Yes | Yes | Yes | Yes | Yes | Yes | Yes | Yes | Yes | Yes | Yes | Yes |
| **Notes:** All specifications are estimated using a Linear Probability Model; This Table presents the results related to our variables of interest from the most inclusive model specifications presented in Figure 4 in the paper and in Tables A, B and C in S4 Appendix; In ‘PV set-up’ we use the smaller sample of mines and a shorter time horizon (2004-2012); In ‘More mines’ results are based on the full sample of mines, for the period 2004-2012; Results in ‘More mines & Longer time horizon’ are based on the full sample of mines for the longer time horizon (2004-2015); We test the robustness of the findings to using alternative spatial and temporal specifications when correcting the standard errors; For each estimated coefficient, we present five sets of standard errors in parentheses: 1) Conley standard errors allowing for spatial correlation within a 1,000 km radius and for infinite serial correlation; 2) Conley standard errors allowing for spatial correlation within a 100 km radius and for infinite serial correlation; 3) Conley standard errors allowing for spatial correlation within a 100 km radius and for 5 years of serial correlation; 4) Conley standard errors allowing for spatial correlation within a 100 km radius and for 1 year of serial correlation; 5) Clustering the standard errors at the level of the territory. | | | | | | | | | | | | |

**Table F. Including 12-month dynamic and spatial conflict lags**

|  | **PV set-up** | | | | **More mines** | | | | **More mines & Longer time horizon** | | | |
| --- | --- | --- | --- | --- | --- | --- | --- | --- | --- | --- | --- | --- |
|  | *Looting* | *Battles* | *Violence* | *Riots* | *Looting* | *Battles* | *Violence* | *Riots* | *Looting* | *Battles* | *Violence* | *Riots* |
|  | (1) | (2) | (3) | (4) | (5) | (6) | (7) | (8) | (9) | (10) | (11) | (12) |
| DF | 0.048^**^ | 0.048^**^ | 0.059^**^ | 0.059^***^ | 0.040^**^ | 0.042^**^ | 0.046^*^ | 0.051^**^ | 0.019 | 0.045^**^ | 0.012 | 0.056^**^ |
|  | (0.021) | (0.021) | (0.026) | (0.021) | (0.018) | (0.020) | (0.027) | (0.021) | (0.015) | (0.021) | (0.030) | (0.024) |
| DF * 3T mines | -0.002 | 0.013 | -0.006 | -0.010 | -0.003 | -0.002 | 0.005 | -0.000 | -0.004 | 0.004 | 0.001 | 0.001 |
|  | (0.014) | (0.021) | (0.022) | (0.012) | (0.005) | (0.006) | (0.009) | (0.007) | (0.003) | (0.005) | (0.008) | (0.008) |
| DF * gold mines | 0.003 | 0.033^***^ | 0.012 | -0.008 | 0.005^***^ | 0.009^***^ | 0.012^***^ | -0.002 | 0.004^***^ | 0.009^***^ | 0.007^***^ | -0.001 |
|  | (0.008) | (0.009) | (0.011) | (0.007) | (0.001) | (0.002) | (0.004) | (0.002) | (0.001) | (0.002) | (0.003) | (0.002) |
|  |  |  |  |  |  |  |  |  |  |  |  |  |
| L1 conflict | 0.023^**^ | 0.109^***^ | 0.095^***^ | 0.016 | 0.027^**^ | 0.109^***^ | 0.092^***^ | 0.017^*^ | 0.020^**^ | 0.102^***^ | 0.082^***^ | 0.021^**^ |
| L2 conflict | 0.036^**^ | 0.078^***^ | 0.047^**^ | -0.004 | 0.030^**^ | 0.082^***^ | 0.044^**^ | -0.003 | 0.033^***^ | 0.074^***^ | 0.041^**^ | -0.000 |
| L3 conflict | 0.018^*^ | 0.033^*^ | 0.040^**^ | 0.014 | 0.012 | 0.028 | 0.037^*^ | 0.014 | 0.016 | 0.038^**^ | 0.033^**^ | 0.002 |
| L4 conflict | 0.018^*^ | 0.030^*^ | 0.049^**^ | 0.010 | 0.011 | 0.034^**^ | 0.047^**^ | 0.011 | 0.005 | 0.030^**^ | 0.044^***^ | 0.008 |
| L5 conflict | 0.007 | 0.034^**^ | 0.039^**^ | -0.009 | 0.000 | 0.036^**^ | 0.036^*^ | -0.009 | 0.020^**^ | 0.022^*^ | 0.032^**^ | 0.012^*^ |
| L6 conflict | -0.014 | -0.019 | 0.002 | 0.015^**^ | -0.015 | 0.006 | 0.001 | 0.016^*^ | -0.005 | 0.020^*^ | 0.024^*^ | 0.011 |
| L7 conflict | -0.011 | 0.052^**^ | 0.035^*^ | 0.000 | -0.013 | 0.044^*^ | 0.033^*^ | 0.000 | -0.014 | 0.039^**^ | 0.026^*^ | 0.014 |
| L8 conflict | -0.007 | 0.006 | -0.025 | -0.004 | -0.008 | 0.003 | -0.027 | -0.003 | -0.004 | 0.024 | -0.012 | -0.004 |
| L9 conflict | -0.001 | 0.010 | 0.019 | 0.007 | 0.006 | 0.013 | 0.017 | 0.006 | 0.007 | 0.010 | 0.018 | 0.009 |
| L10 conflict | 0.011 | 0.007 | 0.019 | -0.004 | 0.009 | 0.007 | 0.016 | -0.004 | 0.004 | 0.029 | 0.017 | 0.002 |
| L11 conflict | -0.000 | 0.018 | 0.008 | -0.009 | -0.013 | 0.022 | 0.006 | -0.009 | 0.011 | 0.031^**^ | 0.022 | -0.009 |
| L12 conflict | -0.012 | 0.009 | -0.030 | -0.019^*^ | -0.019^**^ | 0.017 | -0.033 | -0.019^*^ | -0.014^*^ | 0.024 | -0.004 | -0.013 |
|  |  |  |  |  |  |  |  |  |  |  |  |  |
| adj. terr. conflict | 0.008 | 0.036^**^ | 0.020^**^ | 0.008 | 0.003 | 0.004 | 0.020^**^ | 0.007 | -0.002 | -0.007 | 0.019^***^ | 0.008 |
| L1 adj. terr. conflict | 0.002 | 0.019^*^ | 0.017 | -0.004 | 0.008 | 0.015 | 0.018 | -0.004 | 0.010^*^ | 0.012 | 0.009 | -0.010^***^ |
| L2 adj. terr. conflict | 0.003 | 0.013 | 0.010 | 0.003 | 0.004 | 0.016 | 0.011 | 0.003 | 0.001 | 0.015^*^ | 0.002 | -0.003 |
| L3 adj. terr. conflict | -0.002 | 0.007 | 0.017 | -0.000 | 0.000 | -0.001 | 0.018 | -0.001 | 0.005 | -0.002 | 0.021^**^ | -0.005 |
| L4 adj. terr. conflict | 0.006 | 0.003 | 0.002 | 0.003 | 0.005 | -0.001 | 0.002 | 0.003 | 0.006 | 0.009 | 0.005 | -0.010^**^ |
| L5 adj. terr. conflict | -0.006 | -0.010 | -0.002 | 0.003 | -0.004 | -0.009 | -0.002 | 0.003 | -0.006 | -0.007 | -0.005 | 0.005 |
| L6 adj. terr. conflict | -0.009 | -0.013 | -0.007 | -0.006 | -0.008 | -0.016 | -0.006 | -0.006 | -0.007 | -0.018^**^ | -0.004 | -0.011^**^ |
| L7 adj. terr. conflict | -0.003 | -0.006 | 0.000 | -0.001 | -0.002 | -0.005 | 0.000 | -0.001 | -0.002 | -0.006 | 0.002 | -0.004 |
| L8 adj. terr. conflict | -0.002 | -0.007 | 0.016 | -0.004 | -0.000 | 0.007 | 0.017 | -0.004 | 0.002 | 0.009 | 0.005 | -0.002 |
| L9 adj. terr. conflict | 0.001 | 0.017 | 0.007 | 0.005 | 0.001 | 0.014 | 0.007 | 0.005 | 0.003 | 0.004 | 0.008 | -0.002 |
| L10 adj. terr. conflict | 0.003 | 0.002 | 0.021^*^ | -0.005 | -0.002 | -0.007 | 0.021^*^ | -0.005 | -0.003 | 0.009 | 0.022^**^ | -0.002 |
| L11 adj. terr. conflict | -0.004 | 0.004 | 0.002 | -0.000 | 0.002 | 0.008 | 0.003 | -0.001 | 0.001 | -0.003 | -0.003 | -0.005 |
| L12 adj. terr. conflict | -0.014^**^ | 0.004 | 0.025^*^ | 0.001 | -0.011^*^ | 0.007 | 0.025^*^ | 0.000 | -0.011^**^ | 0.011 | 0.018 | -0.004 |
|  |  |  |  |  |  |  |  |  |  |  |  |  |
| Territory FE | Yes | Yes | Yes | Yes | Yes | Yes | Yes | Yes | Yes | Yes | Yes | Yes |
| Month FE | Yes | Yes | Yes | Yes | Yes | Yes | Yes | Yes | Yes | Yes | Yes | Yes |
| Price controls | Yes | Yes | Yes | Yes | Yes | Yes | Yes | Yes | Yes | Yes | Yes | Yes |
| Weather controls | Yes | Yes | Yes | Yes | Yes | Yes | Yes | Yes | Yes | Yes | Yes | Yes |
|  |  |  |  |  |  |  |  |  |  |  |  |  |
| Observations | 6,720 | 6,720 | 6,720 | 6,720 | 6,720 | 6,720 | 6,720 | 6,720 | 9,240 | 9,240 | 9,240 | 9,240 |
| **Notes:** *** p<0.01, ** p<0.05, * p<0.1; All specifications are estimated using a Linear Probability Model; Conley (1999) standard errors in parentheses, allowing for spatial correlation within a 500 km radius and for infinite serial correlation; Results in Panel A are based on the PV set-up: smaller sample of mines, short time horizon (2004-2012); Results in Panel B are based on the full sample of mines, for the period 2004-2012; Results in Panel C are based on the full sample of mines for the longer time horizon (2004-2015); This Table presents the results from the most inclusive model specifications; We include up to 12-month dynamic and spatial conflict lags. | | | | | | | | | | | | |

**Table G. Conflict intensity**

|  | **PV set-up** | | | | **More mines** | | | | **More mines & Longer time horizon** | | | |
| --- | --- | --- | --- | --- | --- | --- | --- | --- | --- | --- | --- | --- |
|  | *Log nr. Looting* | *Log nr. Battles* | *Log nr. Violence* | *Log nr. Riots* | *Log nr. Looting* | *Log nr. Battles* | *Log nr. Violence* | *Log nr. Riots* | *Log nr. Looting* | *Log nr. Battles* | *Log nr. Violence* | *Log nr. Riots* |
|  | (1) | (2) | (3) | (4) | (5) | (6) | (7) | (8) | (9) | (10) | (11) | (12) |
| DF | 0.062^***^ | 0.065^**^ | 0.137^***^ | 0.037^**^ | 0.045^**^ | 0.059^*^ | 0.115^***^ | 0.029^*^ | 0.018 | 0.071 | 0.054 | 0.044^*^ |
|  | (0.022) | (0.030) | (0.041) | (0.015) | (0.018) | (0.034) | (0.038) | (0.015) | (0.016) | (0.044) | (0.042) | (0.023) |
| DF * 3T mines | -0.008 | 0.001 | -0.014 | -0.014 | -0.002 | 0.004 | 0.004 | -0.001 | -0.005 | 0.012 | -0.005 | -0.000 |
|  | (0.013) | (0.021) | (0.032) | (0.010) | (0.007) | (0.007) | (0.014) | (0.005) | (0.004) | (0.010) | (0.010) | (0.007) |
| DF * gold mines | 0.001 | 0.031^**^ | 0.006 | -0.006 | 0.004^**^ | 0.011^***^ | 0.010^*^ | -0.003 | 0.004^***^ | 0.017^***^ | 0.005 | -0.000 |
|  | (0.006) | (0.015) | (0.013) | (0.005) | (0.002) | (0.004) | (0.005) | (0.002) | (0.001) | (0.005) | (0.004) | (0.002) |
|  |  |  |  |  |  |  |  |  |  |  |  |  |
| Territory FE | Yes | Yes | Yes | Yes | Yes | Yes | Yes | Yes | Yes | Yes | Yes | Yes |
| Month FE | Yes | Yes | Yes | Yes | Yes | Yes | Yes | Yes | Yes | Yes | Yes | Yes |
| Price controls | Yes | Yes | Yes | Yes | Yes | Yes | Yes | Yes | Yes | Yes | Yes | Yes |
| Weather controls | Yes | Yes | Yes | Yes | Yes | Yes | Yes | Yes | Yes | Yes | Yes | Yes |
| Conflict controls | Yes | Yes | Yes | Yes | Yes | Yes | Yes | Yes | Yes | Yes | Yes | Yes |
|  |  |  |  |  |  |  |  |  |  |  |  |  |
| Observations | 7,350 | 7,350 | 7,350 | 7,350 | 7,350 | 7,350 | 7,350 | 7,350 | 9,870 | 9,870 | 9,870 | 9,870 |
| **Notes:** *** p<0.01, ** p<0.05, * p<0.1; All specifications are estimated using a Linear Probability Model; Conley (1999) standard errors in parentheses, allowing for spatial correlation within a 500 km radius and for infinite serial correlation; Results in Panel A are based on the PV set-up: smaller sample of mines, short time horizon (2004-2012); Results in Panel B are based on the full sample of mines, for the period 2004-2012; Results in Panel C are based on the full sample of mines for the longer time horizon (2004-2015); This Table presents the results related to our variables of interest from the most inclusive model specifications; The dependent variables measure conflict intensity; Specifically, we take the natural log of the number of events + 1, such that the dependent variable equals 0 for months without an event. | | | | | | | | | | | | |

**Table H. Combined conflict measure**

|  | **PV set-up** | **More mines** | **More mines & Longer time horizon** |
| --- | --- | --- | --- |
|  | (1) | (2) | (3) |
| DF | 0.068^**^ | 0.063^**^ | 0.048 |
|  | (0.030) | (0.030) | (0.036) |
| DF * 3T mines | -0.001 | -0.000 | 0.003 |
|  | (0.024) | (0.009) | (0.008) |
| DF * gold mines | 0.033^***^ | 0.016^***^ | 0.013^***^ |
|  | (0.012) | (0.004) | (0.004) |
|  |  |  |  |
| Territory FE | Yes | Yes | Yes |
| Month FE | Yes | Yes | Yes |
| Price controls | Yes | Yes | Yes |
| Weather controls | Yes | Yes | Yes |
| Conflict controls | Yes | Yes | Yes |
|  |  |  |  |
| Observations | 7,350 | 7,350 | 9,870 |
| **Notes:** *** p<0.01, ** p<0.05, * p<0.1; All specifications are estimated using a Linear Probability Model; Conley (1999) standard errors in parentheses, allowing for spatial correlation within a 500 km radius and for infinite serial correlation; Results in Panel A are based on the PV set-up: smaller sample of mines, short time horizon (2004-2012); Results in Panel B are based on the full sample of mines, for the period 2004-2012; Results in Panel C are based on the full sample of mines for the longer time horizon (2004-2015); The dependent variable is a conflict indicator that equals one if any of the looting, battles, violence against civilians or riot events occurred within a specific territory and month. | | | |

**Table I. Summary statistics on territory-share of mines**

|  | N | Mean | SD | Min | Max |
| --- | --- | --- | --- | --- | --- |
| Share of 3T mines in DF treatment area | 27 | 0.031 | 0.036 | 0 | 0.115 |
| Share of gold mines in DF treatment area | 27 | 0.029 | 0.035 | 0 | 0.120 |
| Share of 3T mines outside DF treatment area | 43 | 0.004 | 0.012 | 0 | 0.068 |
| Share of gold mines outside DF treatment area | 43 | 0.005 | 0.013 | 0 | 0.058 |
| **Notes:** This Table presents summary statistics for the territory-shares of 3T and gold mines across treated and non-treated territories. | | | | | |

**Table J. Territory-share of 3T and gold mines**

|  | **PV set-up** | | | | **More mines** | | | | **More mines & Longer time horizon** | | | |
| --- | --- | --- | --- | --- | --- | --- | --- | --- | --- | --- | --- | --- |
|  | *Looting* | *Battles* | *Violence* | *Riots* | *Looting* | *Battles* | *Violence* | *Riots* | *Looting* | *Battles* | *Violence* | *Riots* |
|  | (1) | (2) | (3) | (4) | (5) | (6) | (7) | (8) | (9) | (10) | (11) | (12) |
| DF | 0.046^*^ | 0.021 | 0.068^**^ | 0.054^***^ | 0.025 | 0.030 | 0.028 | 0.045^**^ | 0.008 | 0.024 | 0.003 | 0.043^*^ |
|  | (0.024) | (0.030) | (0.030) | (0.019) | (0.018) | (0.030) | (0.032) | (0.020) | (0.016) | (0.035) | (0.037) | (0.023) |
| DF * share 3T mines | -0.075 | -0.004 | -0.097 | -0.370 | -0.238 | -0.065 | 0.437 | -0.170 | -0.359^*^ | 0.414 | 0.126 | -0.039 |
|  | (0.328) | (0.495) | (0.562) | (0.250) | (0.314) | (0.432) | (0.657) | (0.349) | (0.210) | (0.407) | (0.516) | (0.451) |
| DF * share gold mines | 0.118 | 1.523^***^ | 0.530 | -0.240 | 0.766^***^ | 1.726^***^ | 1.963^***^ | -0.453 | 0.653^***^ | 1.955^***^ | 1.157^**^ | -0.061 |
|  | (0.340) | (0.443) | (0.469) | (0.268) | (0.185) | (0.408) | (0.666) | (0.295) | (0.112) | (0.370) | (0.460) | (0.350) |
|  |  |  |  |  |  |  |  |  |  |  |  |  |
| Territory FE | Yes | Yes | Yes | Yes | Yes | Yes | Yes | Yes | Yes | Yes | Yes | Yes |
| Month FE | Yes | Yes | Yes | Yes | Yes | Yes | Yes | Yes | Yes | Yes | Yes | Yes |
| Price controls | Yes | Yes | Yes | Yes | Yes | Yes | Yes | Yes | Yes | Yes | Yes | Yes |
| Weather controls | Yes | Yes | Yes | Yes | Yes | Yes | Yes | Yes | Yes | Yes | Yes | Yes |
| Conflict controls | Yes | Yes | Yes | Yes | Yes | Yes | Yes | Yes | Yes | Yes | Yes | Yes |
|  |  |  |  |  |  |  |  |  |  |  |  |  |
| Observations | 7,350 | 7,350 | 7,350 | 7,350 | 7,350 | 7,350 | 7,350 | 7,350 | 9,870 | 9,870 | 9,870 | 9,870 |
| **Notes:** *** p<0.01, ** p<0.05, * p<0.1; All specifications are estimated using a Linear Probability Model; Conley (1999) standard errors in parentheses, allowing for spatial correlation within a 500 km radius and for infinite serial correlation; Results in Panel A are based on the PV set-up: smaller sample of mines, short time horizon (2004-2012); Results in Panel B are based on the full sample of mines, for the period 2004-2012; Results in Panel C are based on the full sample of mines for the longer time horizon (2004-2015); This Table presents the results related to our variables of interest from the most inclusive model specifications; The DF treatment indicator is interacted with the territory-share of 3T and gold mines, rather than their number. | | | | | | | | | | | | |

**Table K. Territory-specific time trends**

|  | **PV set-up** | | | | **More mines** | | | | **More mines & Longer time horizon** | | | |
| --- | --- | --- | --- | --- | --- | --- | --- | --- | --- | --- | --- | --- |
|  | *Looting* | *Battles* | *Violence* | *Riots* | *Looting* | *Battles* | *Violence* | *Riots* | *Looting* | *Battles* | *Violence* | *Riots* |
|  | (1) | (2) | (3) | (4) | (5) | (6) | (7) | (8) | (9) | (10) | (11) | (12) |
| DF | 0.075^***^ | 0.029 | 0.147^***^ | 0.069^***^ | 0.053^***^ | 0.019 | 0.094^***^ | 0.063^**^ | 0.048^***^ | 0.011 | 0.094^***^ | 0.054^***^ |
|  | (0.025) | (0.028) | (0.039) | (0.024) | (0.020) | (0.032) | (0.031) | (0.026) | (0.018) | (0.030) | (0.034) | (0.021) |
| DF * 3T mines | -0.021^*^ | -0.016 | -0.008 | -0.034^**^ | -0.006 | -0.015 | 0.002 | -0.005 | -0.005 | -0.010 | 0.006 | -0.006 |
|  | (0.012) | (0.019) | (0.024) | (0.015) | (0.006) | (0.010) | (0.011) | (0.006) | (0.005) | (0.010) | (0.011) | (0.005) |
| DF * gold mines | 0.003 | 0.023^***^ | 0.010 | -0.005 | 0.005^**^ | 0.006^*^ | 0.010^*^ | -0.003 | 0.004^**^ | 0.002 | 0.009^*^ | -0.003 |
|  | (0.012) | (0.007) | (0.014) | (0.008) | (0.002) | (0.004) | (0.005) | (0.002) | (0.001) | (0.004) | (0.005) | (0.002) |
|  |  |  |  |  |  |  |  |  |  |  |  |  |
| Territory FE | Yes | Yes | Yes | Yes | Yes | Yes | Yes | Yes | Yes | Yes | Yes | Yes |
| Month FE | Yes | Yes | Yes | Yes | Yes | Yes | Yes | Yes | Yes | Yes | Yes | Yes |
| Price controls | Yes | Yes | Yes | Yes | Yes | Yes | Yes | Yes | Yes | Yes | Yes | Yes |
| Weather controls | Yes | Yes | Yes | Yes | Yes | Yes | Yes | Yes | Yes | Yes | Yes | Yes |
| Conflict controls | Yes | Yes | Yes | Yes | Yes | Yes | Yes | Yes | Yes | Yes | Yes | Yes |
| Terr. time trends | Yes | Yes | Yes | Yes | Yes | Yes | Yes | Yes | Yes | Yes | Yes | Yes |
|  |  |  |  |  |  |  |  |  |  |  |  |  |
| Observations | 7,350 | 7,350 | 7,350 | 7,350 | 7,350 | 7,350 | 7,350 | 7,350 | 9,870 | 9,870 | 9,870 | 9,870 |
| **Notes:** *** p<0.01, ** p<0.05, * p<0.1; All specifications are estimated using a Linear Probability Model; Conley (1999) standard errors in parentheses, allowing for spatial correlation within a 500 km radius and for infinite serial correlation; Results in Panel A are based on the PV set-up: smaller sample of mines, short time horizon (2004-2012); Results in Panel B are based on the full sample of mines, for the period 2004-2012; Results in Panel C are based on the full sample of mines for the longer time horizon (2004-2015); This Table presents the results related to our variables of interest from the most inclusive model specifications; We include territory-specific time trends. | | | | | | | | | | | | |
